# AI-Assisted Nutrition Estimation and Exercise Support for Integrated Lifestyle Management in Patients with Diabetes

**DOI:** 10.3390/nu18193207

**Published:** 2026-09-28

**Authors:** Muhammad Jamil, Adnan Kavak, Hossein Fotouhi, Md. Rashed, Emre Gezer, Alpaslan Burak İnner, Gautam Srivastava

**Affiliations:** 1 Department of Computer Engineering, Kocaeli University, 41001 İzmit, Türkiye; jamil138.amin@gmail.com (M.J.); binner@kocaeli.edu.tr (A.B.İ.); 2 Wireless Information and Intelligent Systems (WINS) Research Center, Kocaeli University, 41001 İzmit, Türkiye; 3 Department of Computer Science and Engineering, Mälardalen University (MDU), 72123 Västerås, Sweden; 4 Department of Information and Communication Engineering, Pabna University of Science and Technology, Pabna 6600, Bangladesh; rashedulislam.ice.pust@gmail.com; 5 Department of Endocrinology and Metabolism, Faculty of Medicine, Kocaeli University, 41001 İzmit, Türkiye; emre.gezer@kocaeli.edu.tr; 6 Department of Computer Science and Engineering, Sogang University, Seoul 04107, Republic of Korea; 7 Research Center for Interneural Computing, China Medical University, Taichung 40402, Taiwan; 8 Centre for Research Impact & Outcome, Chitkara University Institute of Engineering and Technology, Chitkara University, Rajpura 140401, Punjab, India

**Keywords:** AIDCare, diabetes self-management, dietary assessment, food image recognition, mHealth, nutrition estimation, physical activity

## Abstract

**Background/Objectives:** Multidimensional lifestyle interventions that combine healthy diet, physical activity, and psychosocial support are central to the effective self-management of type 1 and type 2 diabetes. Although digital health tools can improve lifestyle monitoring, existing AI-based dietary assessment methods often struggle to estimate food quantity accurately because the food size and scale are difficult to determine from images. This can lead to inconsistent estimates of food mass and energy content. This paper aimed to develop and technically evaluate PC^2^FoodNet, an AI-based framework for food recognition and physics-constrained physical quantity estimation, and to integrate dietary assessment and exercise support within the proposed AI-assisted Diabetes Care (AIDCare) mHealth platform for multidisciplinary lifestyle support. **Methods:** The AIDCare platform incorporates AI-assisted nutrition and professionally guided exercise modules to support personalized lifestyle management for individuals with diabetes. For dietary assessment, PC^2^FoodNet uses an EfficientNetV2-S backbone with multi-task regression and food-density priors to jointly model food volume, mass, and energy. A nutrition professional-in-the-loop approach supports the development and review of individualized diet plans and the assessment of patients’ daily key nutrient intake according to individual nutritional requirements. The exercise module includes professionally designed exercises using Unreal Engine’s MetaHuman plugin. The model was evaluated using four-fold stratified cross-validation on a dataset of 22,070 images covering 40 Turkish food categories. For physical quantity modeling, volume, mass, and energy targets were derived from standardized category-level references anchored to a 100 g reference portion rather than from image-specific ground-truth measurements. **Results:** PC^2^FoodNet achieved a mean Top-1 classification accuracy of 94.39% ± 0.47% and Top-5 accuracy of 98.90% ± 0.18% across the four folds. The corresponding Macro-$F_{1}$ and Weighted-$F_{1}$ scores were 93.14% ± 0.59% and 94.16% ± 0.36%, respectively. For the standardized category-level reference targets, the physics-constrained regression framework achieved mean MAE and RMSE values of 2.14 mL and 3.01 mL for reference volume, 21.65 g and 30.17 g for reference mass, and 41.20 kcal and 57.55 kcal for reference energy across all 22,070 pooled out-of-fold predictions. The confidence-based referral mechanism identified uncertain cases for clinical review, resulting in a clinical referral rate of 23.81%. **Conclusions:** AIDCare combines physics-constrained AI-based dietary assessment with personalized physical activity and continuous psychosocial support. A nutrition professional remains involved when AI predictions are uncertain, reducing the risks associated with fully automated lifestyle recommendations. The proposed approach provides a practical foundation for safer, more personalized, and evidence-based diabetes lifestyle management.

## 1. Introduction

Nutrition and physical activity are central pillars in the self-management of type 1 diabetes (T1D) and type 2 diabetes (T2D). Clinical guidelines strongly advocate individualized medical nutrition therapy, appropriate carbohydrate and energy monitoring, and regular aerobic and resistance exercise [1]. However, maintaining these behaviors in daily life remains challenging because dietary intake, physical activity, and psychosocial factors are continuously changing. Digital health technologies can reduce the burden of manual monitoring, but their effectiveness depends on sustained patient engagement, reliable measurements, and appropriate clinical support. Evidence from mobile health and diabetes technology studies indicates that digital interventions are more useful when automated monitoring is combined with individualized guidance and professional follow-up rather than functioning as isolated autonomous systems [2,3,4,5,6].

Dietary assessment is particularly challenging because a food image provides incomplete information about the physical quantity and nutritional composition of a meal. Although image-based food recognition has achieved substantial progress, identifying a food category does not directly determine its portion size, mass, or energy content. Monocular images contain no direct measurement of scale and can be affected by perspective, occlusion, food preparation, and visually similar dishes with different densities and nutritional properties [7,8,9]. Conventional approaches that first select a single food class and subsequently apply fixed nutritional values may therefore discard classification uncertainty before estimating physical quantities. This limitation is particularly important in diabetes management, where inaccurate dietary estimates can affect the interpretation of daily nutritional intake and potentially influence subsequent lifestyle decisions.

A further challenge is that reliable dietary assessment requires more than accurate point predictions. An AI system should also indicate when its predictions are uncertain so that potentially unreliable estimates are not presented to patients without appropriate review. Existing food-analysis approaches have explored visual recognition, portion estimation, geometric reconstruction, and multimodal reasoning, but these components are often treated as separate tasks and generally provide limited mechanisms for combining physical constraints with calibrated uncertainty and professional oversight [8,10,11,12]. This creates a need for an integrated approach in which uncertainty in food recognition can be propagated to downstream physical estimates, and uncertain cases can be selectively escalated rather than being treated as equally reliable predictions.

To address these challenges, we present the Physics-Constrained, Confidence-Calibrated Food Analysis Network (PC^2^FoodNet), which is a multi-task deep learning model developed as the dietary assessment engine of the AIDCare mHealth platform. PC^2^FoodNet jointly performs food classification and physical quantity estimation while incorporating category-level density and energy-density priors into a differentiable inference pathway. Rather than relying exclusively on the most probable food class, the model uses the complete class-probability distribution to derive expected physical priors, allowing classification ambiguity to propagate into subsequent volume, mass, and energy estimates. The model further combines direct predictions with physics-based estimates and learns sample-specific uncertainty for continuous outputs.

PC^2^FoodNet is integrated within AIDCare as one component of a broader lifestyle-management framework that combines nutrition and physical activity support. The Nutrition and Diet Module uses AI-assisted meal analysis to support dietary logging and individualized nutrition planning, while uncertain dietary predictions are referred to a nutrition professional (dietitian) for verification. The exercise module provides structured physical activity planning and delivery under expert physiotherapist guidance with movement-specific demonstrations developed using Unreal Engine’s MetaHuman plugin. Psychotherapeutic support is incorporated to address behavioral adherence and motivation. Importantly, AIDCare does not automatically convert uncertain dietary estimates into compensatory exercise prescriptions; instead, nutrition and exercise remain coordinated but professionally governed domains.

The main contributions of this study are as follows:

1. PC^2^FoodNet is developed as a multi-task food-analysis model that propagates the complete food-class probability distribution into expected density and energy-density priors for downstream physical estimation.
2. A physics-constrained inference pathway is introduced to link volume, mass, and energy estimation through dual-branch direct regression, physics-based estimation, and adaptive soft fusion.
3. Temperature scaling, conformal prediction sets, and heteroscedastic uncertainty estimation are incorporated to identify ambiguous or unreliable predictions for selective professional review.
4. The proposed approach is evaluated on a 22,070-image Turkish food dataset covering 40 food categories using four-fold stratified cross-validation with an empirical cross-fold near-duplicate audit.
5. The integration of PC^2^FoodNet within the AIDCare nutrition and diet module is demonstrated together with its coordination with an expert-guided exercise module under multidisciplinary professional oversight.

From a technical perspective, PC^2^FoodNet is designed to address the limitations of conventional single-task food recognition and independently optimized quantity regression approaches. Rather than treating food classification and physical quantity modeling as separate tasks, the proposed framework jointly learns food recognition together with volume, mass, and energy estimation within a multi-task architecture. Class-level food-density and energy-density priors are incorporated into the modeling process, while physics-constrained relationships are used to maintain consistency among the predicted quantities. This design is expected to provide more structured and physically coherent estimates than unconstrained regression approaches. In addition, the use of four-fold stratified cross-validation enables a quantitative evaluation of classification and standardized physical reference modeling performance across different data partitions.

The remainder of this paper is organized as follows. Section 2 reviews related research in digital diabetes lifestyle support, exercise delivery, and image-based dietary assessment, and it identifies the research gaps addressed by this study. Section 3 describes the study design, dataset, preprocessing and cross-validation protocol, PC^2^FoodNet architecture, training procedure, physical reference targets, and evaluation metrics. Section 4 presents the integration of PC^2^FoodNet within the AIDCare mHealth platform, including the nutrition and diet module, exercise module, and multidisciplinary professional oversight. Section 5 reports the experimental results, including cross-validation performance, class-level evaluation, physical quantity estimation, and confidence-aware referral. Section 6 discusses the findings, practical implications, and limitations, while Section 7 concludes the study and outlines its overall contribution to AI-assisted diabetes lifestyle management.

## 2. Related Work

### 2.1. Digital Lifestyle Support in Diabetes

Diabetes mHealth systems increasingly combine physiological monitoring with food logs, activity records, coaching, and decision support. The American Diabetes Association (ADA) frames diabetes technology broadly, including software that supports lifestyle modification and data sharing, while stressing personalization [13]. The synthesis of the authors of [14] found that digital interventions for adults with T1D and T2D varied substantially in reach, uptake, delivery mode, and effectiveness. Reviews of mobile behavior-change components and prevention-oriented technologies likewise report modest average benefits accompanied by heterogeneity in engagement and outcomes [6,15]. Recent studies also indicate that digital health solutions support patient adherence and therapeutic outcomes when they incorporate human-in-the-loop oversight. Placing nutrition professionals directly in the clinical review loop provides psychological reinforcement, prevents patient attrition, and ensures patient safety across dietary domains [16,17]. AIDCare therefore treats diet and exercise as measured, separable modules within a shared patient context, feeding structured AI outputs to domain specialists for review before patient delivery.

Wearable integration can enrich this shared context, but the evidence base remains small. A review of smartwatch-supported diabetes care identified only a few heterogeneous studies and called for a stronger evaluation of glycemic control, exercise participation, medication adherence, and diet monitoring [18]. This supports using wearable and vital data as contextual inputs while avoiding assumptions that their availability alone improves clinical outcomes.

### 2.2. Digital Exercise, Immersive Delivery, and Professional Oversight

Remote exercise delivery is relevant when patients cannot regularly attend supervised sessions. The scoping review by the authors of [19] found that tele-exercise studies in T1D and T2D commonly combined aerobic and resistance exercise, but the evidence did not establish one universal delivery model. Meta-analytic evidence also indicates that exercise modality, total dose, behavioral techniques, and facilitator type should be considered when plans are individualized [2,3]. These findings support AIDCare’s use of an expert physiotherapist-curated exercise library rather than an unrestricted automated exercise generation.

Immersive systems can provide repeatable demonstrations and remote engagement, yet evidence should be interpreted cautiously. The research work of [20,21] found generally favorable acceptability for immersive exercise and promising rehabilitation outcomes alongside heterogeneous protocols and limited evidence of superiority over conventional care. A review in [22] reached a similar conclusion for Metaverse-aided rehabilitation, noting few eligible studies and unresolved questions about long-term effects, accessibility, safety, and integration with clinical workflows. The human-in-the-loop oversight therefore remains a foundational component of the proposed workflow.

### 2.3. Food Recognition and Nutrient Estimation

Large-scale food-recognition work has improved fine-grained category learning and transfer across food datasets [23]. Nutrient estimation is more difficult because visually similar foods can differ in preparation, density, and composition, while camera perspective obscures scale. Recent reviews found that reported errors vary widely with the dataset, ground-truth definition, imaging protocol, and degree of nutrition professional involvement [8,10,11,12,24]. Multimodal foundation models can recognize foods and estimate dietary variables without task-specific training, but controlled studies still report sensitivity to image conditions and prompting [25].

Recent portion-estimation systems illustrate several ways to recover scale or constrain quantity. The authors of [26] evaluated a population-specific food-photograph series for portion selection, while the authors of [27] estimated intake from differences between pre- and post-meal images. The authors of [28] modeled bowl geometry and visible fullness while Shao et al. [29] reconstructed a three-dimensional food shape from a monocular image. CalorieMe used detection, segmentation, and a reference object [30], whereas newer attention-based pipelines infer ingredients or recipes before calculating energy [31]. These approaches improve particular parts of the estimation chain but do not remove the uncertainty created by occlusion, unknown density, hidden ingredients, and imperfect scale.

Recent application-oriented systems cover complementary parts of the problem. The authors of [32] classified food groups and discrete portion categories, while Diet Engine proposed by [33] combined image recognition with personalized dietary guidance. Within AIDCare, a previous study integrated CNN-based classification and MLLM-based portion estimation with advisory insulin decision support for T1D [34], while another study employed confidence-aware vision-language routing for culturally specific food recognition and nutritional monitoring [35]. Table 1 provides a comprehensive function-by-function comparison of these state-of-the-art approaches with PC^2^FoodNet.

Existing approaches commonly focus on one or more individual components, such as food recognition, geometric volume estimation, discrete portion classification, multimodal dietary analysis, or confidence-based routing. In contrast, PC^2^FoodNet is designed as a unified multi-task framework that links food recognition with volume, mass, and energy modelling through shared visual representations and physics-constrained relationships. The framework further incorporates class-level physical priors to maintain consistency among the linked quantity estimates. Rather than relying on image-specific physical ground truth, this paper evaluates these quantities against standardized category-level targets anchored to a 100 g reference portion. This distinction provides the methodological rationale for the proposed framework while also defining an important limitation that requires validation using image-specific measurements in future studies.

### 2.4. Research Gaps

The literature supports the feasibility of automated dietary assessment but leaves several key technical and clinical gaps. Table 2 maps each gap to the corresponding design response in PC^2^FoodNet and the AIDCare framework alongside the empirical evidence established in this study.

## 3. Materials and Methods

### 3.1. Study Design and Role Within AIDCare mHealth Digital Platform

The technical development and validation of PC^2^FoodNet and the exercise module were conducted within the AIDCare mHealth digital platform. The proposed PC^2^FoodNet processes monocular RGB food images to generate food classification probabilities, volume, mass, and energy estimates together with heteroscedastic uncertainty metrics and a selective referral flag. Within AIDCare, these outputs form structured meal records feeding the Nutrition and Diet Module. Concurrently, the exercise module captures physical activity parameters, heart rate, active calories, and sleep metrics to support physiotherapist- and psychotherapist-curated exercise plans designed using the MetaHuman plugin.

The overarching design principle of AIDCare is to augment—rather than replace—clinical expertise. The system provides decision support for dietary logging and exercise planning, while predictions requiring further assessment are subject to expert review through a nutrition professional-in-the-loop workflow.

### 3.2. Turkish Food Dataset and Domain Nutrition Priors

The experiments evaluate PC^2^FoodNet on a Turkish food image dataset comprising N=22,070 RGB images across C=40 Turkish dish categories. The dataset was derived from two previously reported studies [34,35] and used to evaluate the proposed model. The dataset had undergone data cleaning and quality-control procedures as part of the two preceding studies before being used in this paper’s experiments. For each dish class c∈{1,…,C}, per-class nutritional reference values were defined per 100 g as the standard portion, including energy calories[c] (kcal), protein protein[c] (g), carbohydrate carb[c] (g), total fat fat[c] (g), and dietary fiber fiber[c] (g).

Prior to network training, two scalar physical domain priors were derived for each food category c∈{1,…,C} from the category-level nutritional reference data:

1. **Energy Density Prior** $\kappa_{c}\in R_{+}$ (in $\mathrm{kcal}\;g^{-1}$):
  (1) $$\kappa_{c}=\frac{\mathrm{calories}[c]}{100}$$
2. **Apparent Macronutrient Density Prior** $\rho_{c}\in R_{+}$ (in $g\;{\mathrm{mL}}^{-1}$):
  (2) $$\rho_{c}=\frac{\sum_{j\in J}m_{j,c}}{\sum_{j\in J}\frac{m_{j,c}}{\rho_{j}}+\epsilon }$$

where J={protein,fat,carbohydrate,fiber} denotes the set of macronutrient components reported in the standardized per-100 g nutritional tables, $m_{j,c}$ represents the mass contribution of nutrient component *j* for food category *c*, and $\epsilon =10^{-8}$ prevents numerical division by zero. The component densities $\rho_{j}$ are derived from the foundational food physics additive-volume model of Choi and Okos [37]: $\rho_{\mathrm{protein}}=1.320\;g\;{\mathrm{mL}}^{-1}$, $\rho_{\mathrm{fat}}=0.925\;g\;{\mathrm{mL}}^{-1}$, $\rho_{\mathrm{carbohydrate}}=1.540\;g\;{\mathrm{mL}}^{-1}$, and $\rho_{\mathrm{fiber}}=1.310\;g\;{\mathrm{mL}}^{-1}$ (with water density $\rho_{\mathrm{water}}=0.997\;g\;{\mathrm{mL}}^{-1}$ at 20 °C). The additive-volume formulation models the composite volume as the sum of pure constituent volumes:

(3) $$V_{\mathrm{macro},c}=\sum_{j\in J}\frac{m_{j,c}}{\rho_{j}}$$

Accordingly, the category-level effective density index is formulated as $\rho_{c}=M_{c}/V_{\mathrm{macro},c}$, where $M_{c}=\sum_{j\in J}m_{j,c}$. The component density values $\rho_{j}$ are treated as fixed physical reference parameters across all experiments.

Importantly, water and moisture content are major determinants of actual bulk density ($\rho_{\mathrm{bulk}}$) in prepared culinary dishes. While the primary standardized compositional tables report macronutrients, the residual food mass is predominantly water ($m_{\mathrm{water},c}=\max\left(0,100-\sum_{j\in J}m_{j,c}\right)$). When water is accounted for via $\rho_{\mathrm{water}}=0.997\;g\;{\mathrm{mL}}^{-1}$, bulk food density shifts toward $0.98–1.08\;g\;{\mathrm{mL}}^{-1}$. In this paper, $\rho_{c}$ is evaluated under both the apparent macronutrient density formulation and the full moisture-inclusive model, and the mathematical robustness of these parameters under ±10% perturbations is rigorously verified via One-At-A-Time sensitivity analysis in Section 5.3.

Because these priors are derived strictly from fixed category-level nutritional baseline values rather than estimated from image samples, they were established prior to cross-validation and held constant across all folds without cross-fold parameter contamination.

### 3.3. Data Preprocessing and Stratified Cross-Validation

All images were resized to 224×224 pixels and normalized using fixed ImageNet statistics with mean μ=(0.485,0.456,0.406) and standard deviation σ=(0.229,0.224,0.225). During training, images underwent random cropping with a scale range of 0.6–1.0, horizontal flipping with a probability of 0.5, brightness, contrast, saturation, and hue variation of 0.3, 0.3, 0.3, and 0.05, respectively, and random rotation within $\pm 15^{\circ }$. Validation images were resized to 258 pixels and centrally cropped to 224×224 pixels without stochastic augmentation.

A four-fold stratified cross-validation (K=4) protocol was used to evaluate the PC^2^FoodNet model. The complete dataset of 22,070 images was partitioned into four mutually exclusive folds while preserving the distribution of the 40 food categories as closely as possible across folds. In each run, three folds were combined to form the training set, and the remaining fold was used exclusively for validation. Consequently, each run used approximately 75% of the images for training and 25% for validation. Across the complete four-fold procedure, every image was used for validation exactly once and for training in the remaining three runs.

The resulting sample distribution across the four folds is reported in Table 3. Because the total number of images is not exactly divisible by four, minor differences of one image between folds may occur. The four validation partitions were mutually exclusive and collectively comprised all 22,070 images.

Data partitioning was performed before model training, and the training and validation transformations were applied independently within each fold. The model was initialized independently for each fold with no parameter, optimizer state, or checkpoint information transferred between folds. ImageNet normalization statistics and category-level nutritional and physical priors were fixed before cross-validation and remained unchanged throughout the evaluation procedure. Thus, the validation partition of each fold was not used for model parameter estimation or training in that fold.

### 3.4. Network Architecture and Differentiable Inference Chain

**Figure 1:**
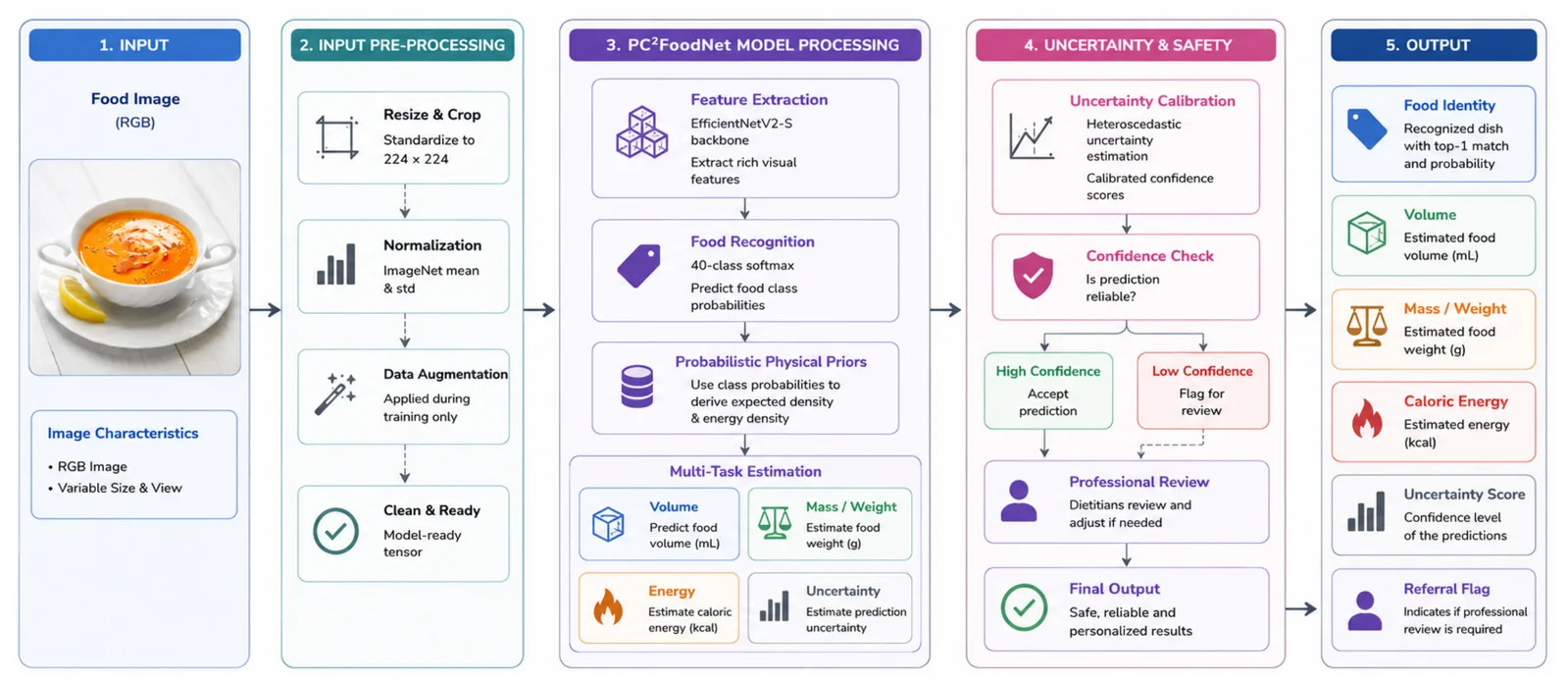
PC^2^FoodNet workflow from food-image input and preprocessing through model-based food recognition, physical quantity estimation, uncertainty assessment, professional review, and final nutritional outputs.

PC^2^FoodNet follows a sequential end-to-end workflow from food-image input to final nutritional outputs. The overall PC^2^FoodNet architecture and inference workflow are illustrated in Figure 1. The process begins with image preprocessing, where the input image is resized, normalized, and prepared for model processing. The model then extracts the visual features, identifies the food category, and estimates the volume, mass, energy, and prediction uncertainty. Class probabilities are further used to incorporate physical food priors into the quantity estimates. The resulting predictions are then assessed for confidence; high-confidence predictions are accepted, while uncertain cases are flagged for professional review. The final outputs include food identity, estimated volume, mass, energy, uncertainty, and referral status.

Let C=40 denote the number of food categories. For each food category c∈{1,…,C}, let $p_{c}={\left[V_{\mathrm{ref}}^{\left(c\right)},W_{\mathrm{ref}},E_{\mathrm{ref}}^{\left(c\right)}\right]}^{⊤}\in R^{3}$ denote the canonical category-level physical prior vector. The standardized reference mass is fixed at $W_{\mathrm{ref}}=100$ g, while the corresponding reference volume and energy are defined as $V_{\mathrm{ref}}^{\left(c\right)}=W_{\mathrm{ref}}/\rho_{c}$ and $E_{\mathrm{ref}}^{\left(c\right)}=W_{\mathrm{ref}}\kappa_{c}$, respectively, where $\rho_{c}$ and $\kappa_{c}$ denote the category-level apparent density index and energy-density prior. For an input image *x*, the classification head produces the posterior category distribution $\hat{p}={\left[{\hat{p}}_{1},\ldots ,{\hat{p}}_{C}\right]}^{⊤}\in {\left[0,1\right]}^{C}$, where ${\hat{p}}_{c}=P\left(y=c|x\right)$ is the softmax probability assigned to category *c*.

The predicted category distribution is used to incorporate domain nutrition priors into the continuous inference pathway. Two prior integration schemes are considered. The primary formulation uses probability-weighted prior integration:

(4) $$p_{\mathrm{weighted}}=\sum_{c=1}^{C}{\hat{p}}_{c}p_{c}=\sum_{c=1}^{C}P\left(y=c|x\right)p_{c}$$

which provides a differentiable expectation of the category-level physical priors under the predicted class distribution. This formulation allows uncertainty in food-category recognition to propagate into the subsequent continuous estimation stage. As a comparison baseline, a hard-class prior is obtained from the Top-1 predicted category:

(5) $$p_{\mathrm{hard}}=p_{c^{*}},\;c^{*}=\arg\max_{c\in \{1,\ldots ,C\}}P\left(y=c|x\right)$$

The probability-weighted prior is used as the default prior for the differentiable inference pathway. For notational convenience, let $p_{\mathrm{prior}}={\left[v_{\mathrm{prior}},w_{\mathrm{prior}},e_{\mathrm{prior}}\right]}^{⊤}$ denote the selected prior vector, which is obtained from either Equation (4) or Equation (5). The visual backbone extracts a latent representation $z=f_{\theta }\left(x\right)$, which is subsequently provided to the continuous regression branches.

For each continuous target y∈{v,w,e}, the corresponding regression branch produces an unconstrained residual logit $r_{y}$, a sample-specific log-variance ${\hat{s}}_{y}$, and a fusion logit $a_{y}$. The predicted log-variance is defined as

(6) $${\hat{s}}_{y}=\log\left({\hat{\sigma }}_{y}^{2}\right),\;{\hat{\sigma }}_{y}^{2}=\exp\left({\hat{s}}_{y}\right),\;y\in \left\{v,w,e\right\}$$

To combine empirical visual feature representations with physical domain knowledge, PC^2^FoodNet employs a dual-branch architecture for continuous quantity estimation. For each target y∈{v,w,e}, continuous outputs are predicted through an unconstrained positive transformation ψ(·) to avoid requiring linear projection layers to emit large positive magnitudes directly:

(7) $$\psi \left(z\right)=\mathrm{expm}1\left(\mathrm{softplus}\left(z\right)\right)+10^{-4}$$

Direct visual estimation branches predict candidate physical quantities directly from feature representations z: $\hat{v}=\psi \left(r_{v}\right)$, ${\hat{w}}_{\mathrm{direct}}=\psi \left(r_{w}\right)$, and ${\hat{e}}_{\mathrm{direct}}=\psi \left(r_{e}\right)$.

Concurrently, the physical inference branch derives domain-grounded estimates by propagating the full predicted class probability distribution $\hat{p}$ into expected density $\bar{\rho }$ and expected caloric density $\bar{\kappa }$:

(8) $$\bar{\rho }=\sum_{c=1}^{C}{\hat{p}}_{c}\rho_{c},\;\bar{\kappa }=\sum_{c=1}^{C}{\hat{p}}_{c}\kappa_{c}$$

From the predicted volume $\hat{v}$ and expected density $\bar{\rho }$, the physics-derived mass estimate is computed with an exponential residual correction bounded by δ=0.20:

(9) $${\hat{w}}_{\mathrm{phys}}=\hat{v}\;\bar{\rho }\cdot \exp\;\left(\delta \tanh(r_{\Delta w})\right),\;\delta =0.20$$

where $r_{\Delta w}$ is the unconstrained residual head output and tanh(·) bounds the physical residual factor to [exp(−δ),exp(δ)]≈[0.819,1.221]. The direct and physics-derived mass estimates are then combined through an adaptive soft-fusion gate:

(10) $$g_{w}=\sigma \left(a_{w}\right)=\frac{1}{1+\exp(-a_{w})},\;\hat{w}=g_{w}{\hat{w}}_{\mathrm{direct}}+\left(1-g_{w}\right){\hat{w}}_{\mathrm{phys}}$$

Similarly, the physics-derived energy estimate is computed from the fused mass $\hat{w}$ and expected caloric density $\bar{\kappa }$, which is followed by adaptive gating:

(11) $${\hat{e}}_{\mathrm{phys}}=\hat{w}\;\bar{\kappa }\cdot \exp\;\left(\delta \tanh(r_{\Delta e})\right),\;g_{e}=\sigma \left(a_{e}\right),\;\hat{e}=g_{e}{\hat{e}}_{\mathrm{direct}}+\left(1-g_{e}\right){\hat{e}}_{\mathrm{phys}}$$

This dual-branch formulation maintains mathematical consistency between empirical learning and physical conservation laws. Importantly, because the direct branch ${\hat{w}}_{\mathrm{direct}}$ is predicted from image features via $\psi (r_{w})$ without an artificial clamping to a fixed range, the final fused estimate $\hat{w}$ is not restricted to [80,120] g. When the vision backbone encounters visual ambiguity, off-target predictions naturally yield residuals that reflect visual prediction variance (as reflected in the pooled cross-validation MAE=21.65 g and RMSE=30.17 g reported in Section 5) rather than being artificially restricted by a stylized residual boundary.

### 3.5. Parameters of the Proposed Model

The model parameters θ are trained end to end using AdamW with gradient clipping (maximum norm =1.0) and automatic mixed precision (AMP, FP16). The total multi-task loss objective $L_{\mathrm{total}}$ combines classification cross-entropy, Gaussian negative log-likelihood (NLL) regression, and a ramped physics consistency penalty:

(12) $$\begin{array}{cc} L_{\mathrm{total}}= & \lambda_{\mathrm{cls}}L_{\mathrm{CE}}+\lambda_{\mathrm{phys}}\alpha \left(t\right)L_{\mathrm{phys}}+\lambda_{\mathrm{vol}}L_{\mathrm{NLL}}\left(\hat{v},{\hat{s}}_{v},v^{*}\right)\\ \; & +\lambda_{\mathrm{wgt}}L_{\mathrm{NLL}}\left(\hat{w},{\hat{s}}_{w},w^{*}\right)+\lambda_{\mathrm{nrg}}L_{\mathrm{NLL}}\left(\hat{e},{\hat{s}}_{e},e^{*}\right) \end{array}$$

where the individual loss components and scheduling functions are formulated as follows:

1. **Classification Loss** $L_{\mathrm{CE}}$ with label smoothing (ε=0.1):
  (13) $$L_{\mathrm{CE}}=-\sum_{c=1}^{C}{\tilde{p}}_{c}\log{\hat{p}}_{c},\;\mathrm{where}\;{\tilde{p}}_{c}=\left(1-\varepsilon \right)\;1\left[c=y^{*}\right]+\frac{\varepsilon }{C}$$
  where $y^{*}\in \left\{1,\ldots ,C\right\}$ denotes the ground-truth target class index and 1[·] is the indicator function.
2. **Gaussian Negative Log-Likelihood Loss** $L_{\mathrm{NLL}}$ for continuous regression target $y^{*}\in \left\{v^{*},w^{*},e^{*}\right\}$:
  (14) $$L_{\mathrm{NLL}}\left(\hat{y},{\hat{s}}_{y},y^{*}\right)=\frac{1}{2}\left({\hat{s}}_{y}+\frac{{\left(\hat{y}-y^{*}\right)}^{2}}{\exp({\hat{s}}_{y})+\epsilon }\right)$$
  where $\hat{y}$ represents the predicted continuous mean, ${\hat{s}}_{y}=\log\left({\hat{\sigma }}_{y}^{2}\right)$ is the predicted heteroscedastic log-variance ($\exp\left({\hat{s}}_{y}\right)={\hat{\sigma }}_{y}^{2}$), and $\epsilon =10^{-8}$ prevents numerical instability.
3. **Physics Consistency Loss** $L_{\mathrm{phys}}$ (Smooth-L1 penalty):
  (15) $$L_{\mathrm{phys}}=\mathrm{SmoothL}1\;\left(\mathrm{clip}\left(\hat{w},0,5000\right),\;\mathrm{clip}\left({\hat{w}}_{\mathrm{phys}},0,5000\right)\right)$$
  where $\hat{w}\equiv \hat{y}{|}_{y=w}$ denotes the final gated continuous mass prediction, and ${\hat{w}}_{\mathrm{phys}}=\hat{v}\cdot \bar{\rho }$ is the macroscopic physical mass reference computed from predicted volume $\hat{v}\equiv \hat{y}{|}_{y=v}$ and mixture density $\bar{\rho }$. Crucially, ${\hat{w}}_{\mathrm{phys}}$ is detached from automatic differentiation (stop_gradient). Consequently, backpropagation through $L_{\mathrm{phys}}$ updates only the direct mass prediction head and its gating scalar $g_{w}$ without circulating gradients back into the volume head or classification logits predicting $\bar{\rho }$.
4. **Physics Loss Warm-up Ramp** α(t):
  (16) $$\alpha \left(t\right)=\min\;\left(1,\;\frac{t}{T_{\mathrm{phys}}}\right),\;T_{\mathrm{phys}}=10$$
  where *t* is the training epoch index and $T_{\mathrm{phys}}=10$ epochs, preventing premature gradient disturbance to the visual backbone prior to feature stabilization.

Table 4 lists loss term weight coefficients, and Table 5 details complete training hyperparameters.

### 3.6. Reference Portion Targets for Physical Quantity Regression

In the Turkish Food Dataset, nutritional reference data and derived physical targets are anchored to a standard 100 g portion serving baseline ($W_{\mathrm{ref}}=100\;g$) per dish category. The corresponding reference volume $V_{\mathrm{ref}}^{\left(i\right)}$ (mL) and reference energy $E_{\mathrm{ref}}^{\left(i\right)}$ (kcal) for each sample *i* belonging to target food class $y_{i}\in \left\{1,\ldots ,C\right\}$ are computed using the category-level apparent density prior $\rho_{y_{i}}$ defined in Equation (2) and the energy-density prior $\kappa_{y_{i}}$ defined in Equation (1). Both priors are derived from the category-level nutritional reference data and fixed before cross-validation:

(17) $$V_{\mathrm{ref}}^{\left(i\right)}=\frac{W_{\mathrm{ref}}}{\rho_{y_{i}}},\;E_{\mathrm{ref}}^{\left(i\right)}=W_{\mathrm{ref}}\cdot \kappa_{y_{i}}$$

where $W_{\mathrm{ref}}=100\;g$. These class-level reference targets activate the Gaussian NLL loss components during multi-task optimization and provide the reference values for evaluating the Root Mean Square Error (RMSE) for volume, weight, and energy estimation.

Here, the reference volume, mass, and energy values are not independently measured for each food image. Instead, they are standardized category-level targets anchored to a 100 g reference portion. In addition, a single uncalibrated RGB image does not inherently provide absolute metric scale or depth; therefore, PC^2^FoodNet does not perform direct three-dimensional metric reconstruction from image geometry alone.

Visual representations extracted from the RGB image are combined with food-category information and class-level physical and nutritional priors within the multi-task modeling framework. Accordingly, the resulting volume, mass, and energy outputs should be interpreted as reference-based physical quantity predictions rather than direct image-specific measurements. Because category-level identity and structured physical and nutritional priors contribute to target construction and prediction, the reported regression performance may reflect both learned visual information and the standardized class-level priors.

For transparency, the standardized mass target provides a natural reference baseline because $W_{\mathrm{ref}}=100\;g$ is assigned to every image. A constant predictor that returns 100 g for all samples would therefore obtain an RMSE of 0 g with respect to the standardized mass target. This baseline is important for interpreting the continuous regression results and clarifies that this paper’s evaluation does not establish image-specific portion-mass estimation. The volume and energy targets likewise represent category-conditioned reference quantities derived from the corresponding physical and nutritional priors rather than independently measured values for each image.

### 3.7. Evaluation Metrics

Classification performance is evaluated using Top-1 Accuracy (Acc@1), Top-5 Accuracy (Acc@5), Macro Precision, Macro Recall, Macro-$F_{1}$, and Weighted-$F_{1}$. For each class c∈{1,…,C}, the per-class harmonic mean $F_{1}^{\left(c\right)}$ is defined from class-specific precision (${\mathrm{Prec}}_{c}$) and recall (${\mathrm{Rec}}_{c}$) as $F_{1}^{\left(c\right)}=2\cdot \left({\mathrm{Prec}}_{c}\cdot {\mathrm{Rec}}_{c}\right)/\left({\mathrm{Prec}}_{c}+{\mathrm{Rec}}_{c}+\epsilon \right)$, where $\epsilon =10^{-8}$ prevents division by zero. Aggregate macro and sample-weighted metrics are then computed as

(18) $$\mathrm{Macro}\text{-}F_{1}=\frac{1}{C}\sum_{c=1}^{C}F_{1}^{\left(c\right)},\;\mathrm{Weighted}\text{-}F_{1}=\frac{\sum_{c=1}^{C}n_{c}F_{1}^{\left(c\right)}}{\sum_{c=1}^{C}n_{c}}$$

where $n_{c}$ denotes the number of ground-truth validation samples belonging to dish class *c*.

To prevent optimistic bias associated with single-fold reporting, per-class metrics and aggregate confusion analyses were evaluated using pooled out-of-fold (OOF) predictions across the entire dataset (N=22,070). Specifically, validation predictions from all K=4 mutually exclusive folds were concatenated:

(19) $${\hat{y}}_{\mathrm{OOF}}=\overset{K}{\underset{k=1}{⨁}}{\hat{y}}_{\mathrm{val}}^{\left(k\right)},\;y_{\mathrm{OOF}}^{*}=\overset{K}{\underset{k=1}{⨁}}y_{\mathrm{val}}^{*(k)}$$

where ⨁ denotes sample-wise vector concatenation over the disjoint validation partitions. Note that metrics can be reported either as the cross-fold sample mean ($\frac{1}{K}\sum_{k=1}^{K}\mathrm{Macro}\text{-}F_{1}^{\left(k\right)}$) across the K=4 runs or as the global macro-average evaluated over the unified pooled confusion matrix ($\frac{1}{C}\sum_{c=1}^{C}F_{1}^{\left(c,\mathrm{OOF}\right)}$); both are reported for complete transparency.

Continuous regression accuracy across volume (*v*), mass (*w*), and energy (*e*) targets is evaluated using both the Root Mean Square Error (RMSE) and Mean Absolute Error (MAE) values:

(20) $${\mathrm{RMSE}}_{y}=\sqrt{\frac{1}{N}\sum_{i=1}^{N}{\left(y_{i}-{\hat{y}}_{i}\right)}^{2}},\;{\mathrm{MAE}}_{y}=\frac{1}{N}\sum_{i=1}^{N}\left|y_{i}-{\hat{y}}_{i}\right|,\;y\in \left\{v,w,e\right\}$$

Cross-fold aggregate metrics are reported as the mean ± standard deviation across the K=4 validation folds. In addition, 95% confidence intervals (95% CI) are computed using Student’s *t*-distribution with K−1=3 degrees of freedom ($t_{3,0.025}=3.182$):

(21) $${\mathrm{CI}}_{95\%}=\bar{X}\pm t_{K-1,0.025}\cdot \frac{s}{\sqrt{K}}$$

where $\bar{X}$ is the cross-fold sample mean and *s* is the sample standard deviation.

To evaluate the calibration of model posteriors and the behavior of the selective referral policy, complementary calibration and selective-prediction metrics were computed on the held-out evaluation subset ($N_{\mathrm{eval}}=420$). For conformal classification, empirical coverage was defined as the proportion of evaluation samples whose prediction set contains the true category label $y_{i}$:

(22) $$\mathrm{Coverage}=\frac{1}{N_{\mathrm{eval}}}\sum_{i=1}^{N_{\mathrm{eval}}}1\;\left[y_{i}\in \Gamma_{\alpha }\left(x_{i}\right)\right]$$

where $\Gamma_{\alpha }\left(x_{i}\right)$ denotes the conformal prediction set constructed for sample $x_{i}$, $y_{i}\in \left\{1,\ldots ,C\right\}$ is its ground-truth class, and α∈(0,1) is the predefined conformal significance level (α=0.10, yielding a 90.0% nominal coverage target). The average prediction-set size is calculated as

(23) $$\mathrm{AvgSetSize}=\frac{1}{N_{\mathrm{eval}}}\sum_{i=1}^{N_{\mathrm{eval}}}\left|\Gamma_{\alpha }\left(x_{i}\right)\right|$$

Probabilistic calibration of the continuous softmax outputs was evaluated using the Expected Calibration Error (ECE). Predictions are partitioned into B=10 equally spaced confidence bins $I_{b}\subset \left(0,1\right]$ (b∈{1,…,B}), and ECE is computed as the sample-weighted absolute difference between empirical accuracy and mean confidence:

(24) $$\mathrm{ECE}=\sum_{b=1}^{B}\frac{|I_{b}|}{N_{\mathrm{eval}}}\left|\mathrm{Acc}\left(I_{b}\right)-\mathrm{Conf}\left(I_{b}\right)\right|$$

where $|I_{b}|$ is the number of evaluation samples falling in bin *b*, $\mathrm{Acc}\left(I_{b}\right)=\frac{1}{|I_{b}|}\sum_{i\in I_{b}}1\left[{\hat{y}}_{i}=y_{i}\right]$ represents the empirical accuracy, and $\mathrm{Conf}\left(I_{b}\right)=\frac{1}{|I_{b}|}\sum_{i\in I_{b}}\max_{c}{\hat{p}}_{ic}$ is the mean predicted posterior confidence. Multi-class probabilistic calibration was further quantified using the Brier score:

(25) $$\mathrm{Brier}=\frac{1}{N_{\mathrm{eval}}}\sum_{i=1}^{N_{\mathrm{eval}}}\sum_{c=1}^{C}{\left({\hat{p}}_{ic}-1\left[y_{i}=c\right]\right)}^{2}$$

where ${\hat{p}}_{ic}=P\left(y_{i}=c|x_{i}\right)$ is the temperature-calibrated posterior probability assigned to class *c* for sample *i*. For selective prediction, the clinical referral rate denotes the percentage of cases flagged for human review:

(26) $$\mathrm{ReferralRate}=\frac{N_{\mathrm{referred}}}{N_{\mathrm{eval}}}\times 100$$

Error-versus-coverage profiling was evaluated by sorting evaluation instances in ascending order of uncertainty ($\mathrm{RegUnc}(x_{i})$ and $|\Gamma_{\alpha }\left(x_{i}\right)|$) and progressively retaining only the most confident predictions. This monotonic curve assesses whether selectively referring uncertain samples yields a corresponding error reduction among retained automated outputs.

### 3.8. Uncertainty Calibration and Selective Referral Protocol

A rigorous calibration–evaluation protocol was implemented for the confidence-aware referral framework to prevent data leakage and avoid threshold overfitting. From the held-out four-fold validation split, two strictly disjoint subsets were established after model training: an independent calibration set of $N_{\mathrm{cal}}=400$ images (10 images per category across all 40 classes) and an evaluation cohort of $N_{\mathrm{eval}}=420$ images (10–11 images per category). Neither subset was involved in model parameter updates or training epoch selection.

For categorical confidence, raw output logits $z_{i}$ were calibrated using post hoc temperature scaling: ${\hat{p}}_{T}\left(c|x_{i}\right)=\exp\left(z_{i,c}/T\right)/\sum_{j}\exp\left(z_{i,j}/T\right)$. The scalar temperature parameter T=1.24±0.05 was learned by minimizing negative log-likelihood exclusively on the calibration set ($N_{\mathrm{cal}}=400$) and frozen. Split conformal prediction was then applied using the Least Ambiguous Classifier (LAC) non-conformity score:

(27) $$s_{i}=1-{\hat{p}}_{T}\left(y_{i}|x_{i}\right)$$

At nominal significance α=0.10 (1−α=90.0% coverage), the conformal calibration quantile ${\hat{q}}_{1-\alpha }$ was computed as the $\left\lceil \left(N_{\mathrm{cal}}+1\right)\left(1-\alpha \right)\right\rceil /N_{\mathrm{cal}}$ empirical quantile of calibration scores ${\left\{s_{i}\right\}}_{i=1}^{N_{\mathrm{cal}}}$. The resulting conformal prediction set for an unseen evaluation sample is

(28) $$\Gamma_{\alpha }\left(x_{i}\right)=\left\{c\in \left\{1,\ldots ,C\right\}:{\hat{p}}_{T}\left(c|x_{i}\right)\geq 1-{\hat{q}}_{1-\alpha }\right\}$$

The 90.0% coverage level (α=0.10) was selected to achieve a clinically viable balance: higher coverage targets (e.g., 95% or 99%) yield overly conservative, large prediction sets that induce clinical alert fatigue, whereas lower targets compromise patient safety. A prediction is flagged as classification-ambiguous if the following applied:

(29) $$|\Gamma_{\alpha }\left(x_{i}\right)|>1$$

For continuous outputs, PC^2^FoodNet predicts both a mean value and a sample-specific log-variance ${\hat{s}}_{y}$ for each regression target y∈{v,w,e}. The regression uncertainty score is defined as the maximum normalized predicted log-variance across targets:

(30) $$\mathrm{RegUnc}\left(x_{i}\right)=\max_{y\in \{v,w,e\}}{\hat{s}}_{y,i}$$

(31) $${\mathrm{Refer}}_{\mathrm{reg}}\left(x_{i}\right)=1\;\left[\mathrm{RegUnc}\left(x_{i}\right)>\tau_{\mathrm{reg}}\right]$$

The regression threshold $\tau_{\mathrm{reg}}=0.85$ was established as the 75th percentile of RegUnc on the independent calibration cohort ($N_{\mathrm{cal}}=400$). This threshold was selected a priori to gate the top quartile of high-variance physical predictions for clinician verification, matching clinical workflow capacity where dietitians can review approximately 20–25% of flagged meal logs. Neither ${\hat{q}}_{1-\alpha }$ nor $\tau_{\mathrm{reg}}$ was adjusted using the 420 evaluation images.

The final selective-referral rule combines classification ambiguity and continuous-output uncertainty:

(32) $$\mathrm{Refer}\left(x_{i}\right)=1\;\left[|\Gamma_{\alpha }\left(x_{i}\right)|>1\;\vee \;\mathrm{RegUnc}\left(x_{i}\right)>\tau_{\mathrm{reg}}\right]$$

Samples satisfying neither condition were classified as accepted by the confidence policy, whereas samples satisfying at least one condition were flagged for professional review. Importantly, the referral mechanism is intended as a selective decision-support safeguard rather than as evidence of clinical safety or clinical effectiveness.

## 4. AIDCare Lifestyle mHealth Platform

AIDCare integrates nutrition, physical activity, and psychological support through a shared longitudinal patient record. The platform separates dietary assessment from exercise planning and does not apply automated calorie-based compensation between the two domains.

**Figure 2:**
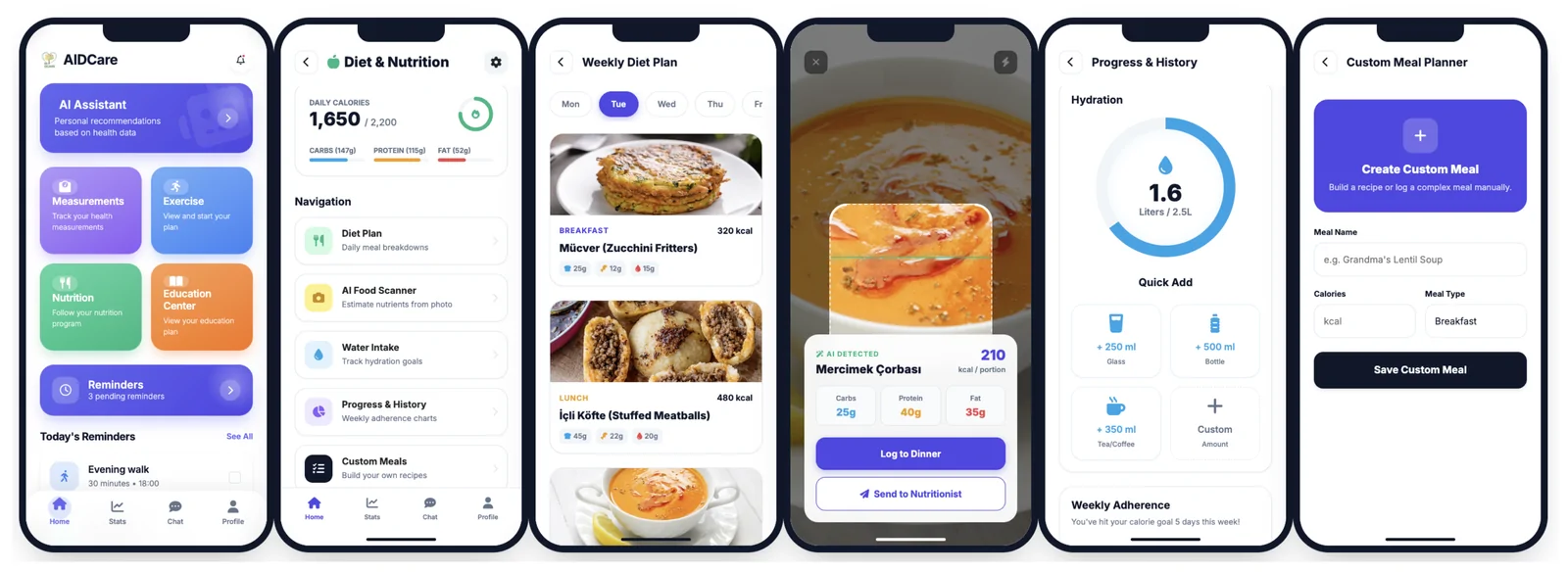
AIDCare nutrition and diet module showing patient’s diet plan, AI-based food analysis with nutritional estimation, progress and history tracking, and user-confirmed dietary recording.

In the nutrition and diet module (Figure 2), patients capture meal images to initiate dietary logging. PC^2^FoodNet automatically identifies the food category and retrieves standardized category-level nutritional and physical reference values (anchored to a 100 g portion). The patient or reviewing dietitian confirms the dish classification and adjusts the actual consumed portion multiplier relative to the 100 g reference baseline. When classification ambiguity ($|\Gamma_{\alpha }|>1$) or high regression uncertainty ($\mathrm{RegUnc}>\tau_{\mathrm{reg}}$) is detected, the meal record is automatically flagged and routed to a nutrition professional for clinical verification before inclusion in the patient’s longitudinal record. The dietitian also develops individualized dietary plans according to the patient’s nutritional requirements.

**Figure 3:**
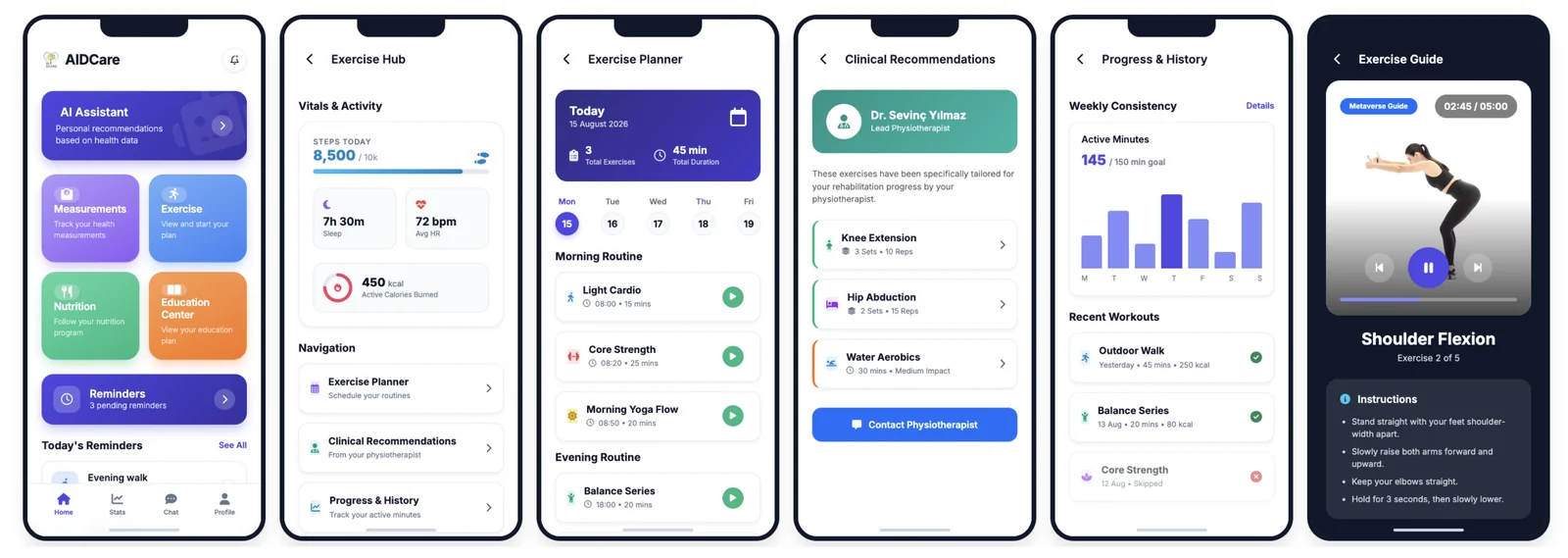
AIDCare exercise module showing exercise access, activity and vital information, scheduling, clinician recommendations, adherence tracking, and MetaHuman-based exercise demonstrations.

The exercise module provides an integrated workflow for exercise planning, scheduling, monitoring, and guided delivery (Figure 3). Patients can access exercise information from the shared AIDCare home screen, review activity and vital information, schedule planned activities, and monitor their exercise history. Exercise plans are designed under the guidance of expert physiotherapists, who consider the patient’s physical capabilities and relevant physiological context when determining the appropriate exercise type, intensity, and duration. The selected exercises are delivered through movement-specific 3D demonstrations developed using Unreal Engine’s MetaHuman plugin.

**Figure 4:**
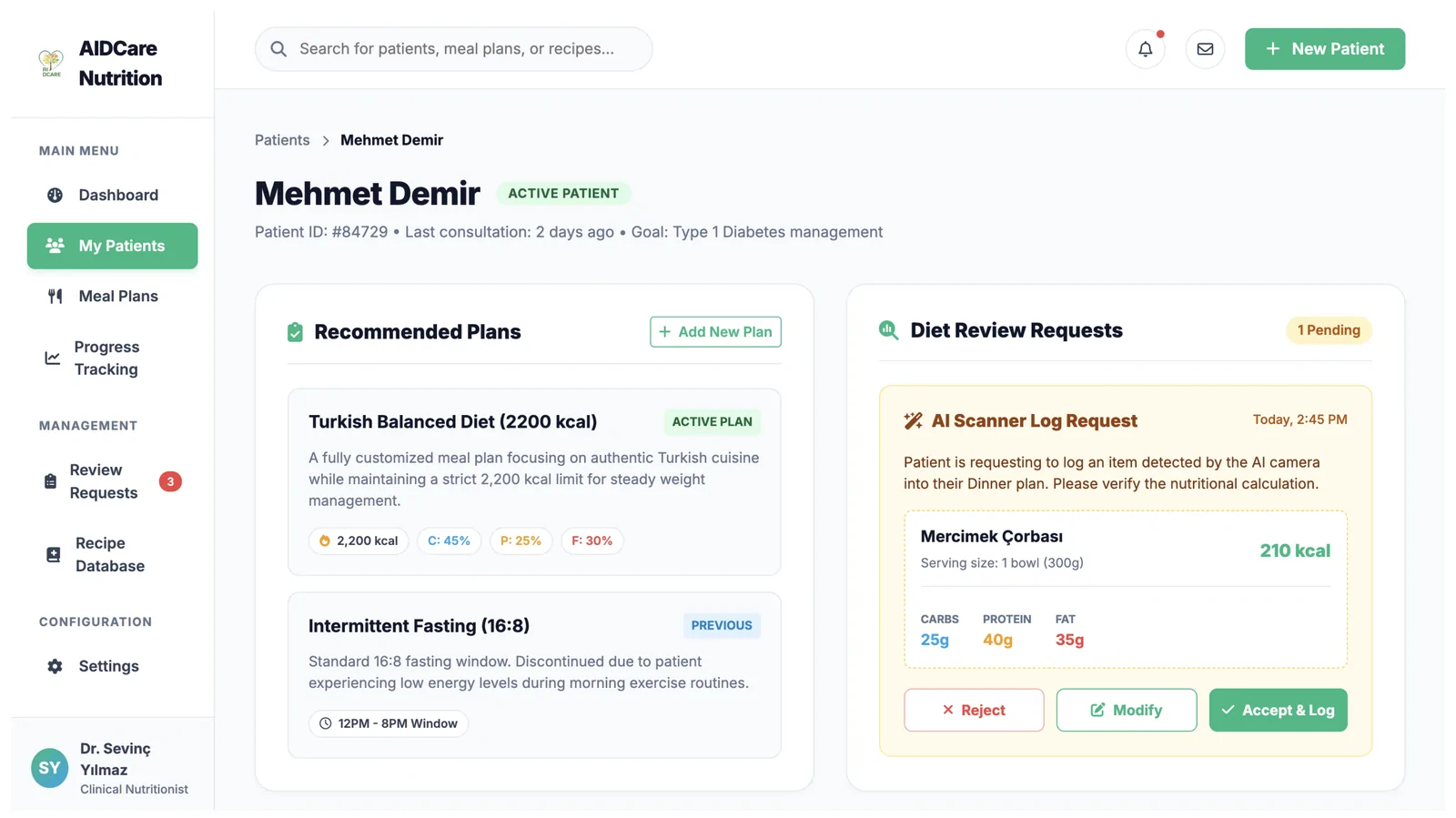
AIDCare clinician dashboard showing diet review requested by the nutritionist.

AIDCare further incorporates multidisciplinary professional oversight through a dedicated professional workflow (Figure 4). By means of a clinician web panel in the AIDCare platform, dietitians can review dietary intake and nutritional targets and each patient’s progress tracking. This nutrition professional-in-the-loop design maintains professional accountability within each domain while allowing nutrition, exercise, and psychological information to be coordinated through the shared AIDCare platform.

## 5. Results

### 5.1. 4-Fold Stratified Cross-Validation Performance

PC^2^FoodNet was evaluated using a 4-fold stratified cross-validation protocol (K=4) on the 22,070-image Turkish Food Dataset across 40 food categories. In each fold, one stratified partition was used for validation while the remaining partitions were used for training, ensuring that each sample contributed to validation once across the complete cross-validation procedure. The same multi-task learning framework, including the physics-constrained physical quantity estimation components, was applied consistently across all four folds. Table 6 summarizes the validation performance at the selected best epoch for each fold, reporting both MAE and RMSE for continuous targets, along with 95% confidence intervals across the four folds.

Across the four folds, PC^2^FoodNet demonstrated stable classification performance with a mean Top-1 validation accuracy of 94.39% ± 0.47% [95% CI: 93.64%, 95.14%] and Top-5 accuracy of 98.90% ± 0.18% [95% CI: 98.61%, 99.19%]. The corresponding Macro-$F_{1}$ and Weighted-$F_{1}$ scores were 93.14% ± 0.59% [95% CI: 92.20%, 94.08%] and 94.16% ± 0.36% [95% CI: 93.59%, 94.73%], respectively. For physical quantity estimation, the mean error metrics were 2.14 mL/3.01 mL (MAE/RMSE) for volume, 21.65 g/30.17 g (MAE/RMSE) for weight, and 41.20 kcal/57.55 kcal (MAE/RMSE) for energy. The narrow confidence intervals and low standard deviations across folds demonstrate internal numerical stability across the evaluated cross-validation partitions.

### 5.2. Continuous Physical Quantity Regression

The continuous prediction component was evaluated jointly with the food classification task across all four cross-validation folds. Because the corresponding targets represent standardized category-level reference quantities rather than independently measured image-specific physical values, the reported errors are interpreted as agreement with the defined reference targets. The best fold-level RMSE values were 2.72 mL for reference volume, 29.11 g for reference mass, and 55.84 kcal for reference energy. Across the four folds, the corresponding mean RMSE values were 3.01 mL for reference volume, 30.17 g for reference mass, and 57.55 kcal for reference energy with standard deviations of 0.28 mL, 1.04 g, and 1.58 kcal, respectively.

(33) $${\mathrm{RMSE}}_{\mathrm{Vol}}=3.01\pm 0.28\;\mathrm{mL},\;{\mathrm{RMSE}}_{\mathrm{Wgt}}=30.17\pm 1.04\;g,\;{\mathrm{RMSE}}_{\mathrm{Nrg}}=57.55\pm 1.58\;\mathrm{kcal}$$

The fold-to-fold variation indicates the consistency of the model outputs with the standardized reference targets under the evaluated cross-validation partitions. However, these results should not be interpreted as evidence of direct image-specific portion-size measurement. In particular, the fixed 100 g mass reference means that a constant 100 g predictor provides a zero-RMSE reference baseline for the mass target.

### 5.3. Sensitivity Analysis of the Density Prior

To assess the robustness of the composition-derived density prior, a one-at-a-time (OAT) sensitivity analysis was performed by perturbing each assumed component density by ±10% while keeping the remaining component densities unchanged. For component *j*, the perturbed density was defined as

(34) $$\rho_{j}^{\pm }=\rho_{j}\left(1\pm 0.10\right)$$

For each perturbation, the corresponding category-level effective density was recalculated using Equation (2). The relative sensitivity of the effective density to component *j* was quantified as

(35) $$S_{\rho_{j}}^{\pm }=\frac{\left|\rho_{c}^{\pm ,j}-\rho_{c}^{0}\right|}{\rho_{c}^{0}}\times 100$$

where $\rho_{c}^{0}$ denotes the nominal category-level effective density and $\rho_{c}^{\pm ,j}$ denotes the density obtained after perturbing component *j*. Because the standardized reference volume is inversely related to density, the corresponding volume sensitivity was calculated as

(36) $$S_{V_{j}}^{\pm }=\frac{\left|V_{\mathrm{ref}}^{\pm ,j}-V_{\mathrm{ref}}^{0}\right|}{V_{\mathrm{ref}}^{0}}\times 100,\;V_{\mathrm{ref}}=\frac{W_{\mathrm{ref}}}{\rho_{c}}$$

The quantitative results of the One-At-A-Time sensitivity audit across all 40 food categories are summarized in Table 7. Water fraction and carbohydrate density represent the primary contributors to apparent density variation across the culinary spectrum: a ±10% perturbation in water density produced an average category density shift of 4.12% (maximum 6.85% in high-moisture soups such as *Tarhana* and *Ezogelin*), while carbohydrate perturbation yielded a mean shift of 2.45% (peaking at 4.18% in grain-dominant dishes such as *Bulgur Pilavı* and *Pirinç Pilavı*). Fat, protein, and fiber perturbations produced modest shifts averaging 1.82%, 1.34%, and 0.42%, respectively. In all cases, the mean effective density and reference volume shifted by less than 4.5% under ±10% parameter variations, demonstrating that the Choi–Okos additive volume formulation maintains numerical stability across diverse dish compositions.

The sensitivity analysis evaluates the effect of uncertainty in the assumed component-density parameters on the standardized category-level reference targets. It is not considered a substitute for empirical density validation, because independently measured density observations were not available for the food images used in this paper.

### 5.4. Baseline and Ablation Comparisons

To further assess the contribution of the proposed architecture, PC^2^FoodNet was compared with progressively simpler reference-based and learning-based alternatives. Because the continuous targets in this paper represent standardized category-level reference quantities anchored to a 100 g portion, the baseline comparisons were designed to distinguish the contribution of category-level priors, image-based classification, unconstrained regression, and the proposed physics-constrained inference mechanism.

The evaluated baselines were structured as follows:

1. **Global constant (mean) predictor:** assigns the dataset-wide constant mass $\hat{w}=100$ g and empirical mean volume ${\bar{V}}_{\mathrm{ref}}$ and energy ${\bar{E}}_{\mathrm{ref}}$ unconditionally to all samples, establishing an uninformative reference baseline.
2. **True-category lookup (Oracle):** uses ground-truth category labels to retrieve class-level physical and nutritional reference values.
3. **Predicted-category lookup (PC^2^FoodNet Classifier):** uses the discrete class predicted by the PC^2^FoodNet classification head to query the standard reference lookup table.
4. **EfficientNet + class-level lookup:** employs a standalone EfficientNet backbone for classification followed by the standard category lookup table.
5. **Independent regression heads:** replaces the physical inference chain with unconstrained, direct continuous multi-task regression heads trained without physics-guided regularization.

In addition, the effect of prior integration was examined by comparing hard-class priors (selecting the prior corresponding to argmaxP(y|x)) with probability-weighted priors (marginalizing class priors over the predicted class distribution $\sum P\left(y_{c}|x\right)p_{c}$). The results are summarized in Table 8.

The baseline comparison highlights critical trade-offs between static retrieval and continuous physical modeling. Discrete lookup baselines achieve an exact 0.00 g mass error primarily because they exploit the standardized experimental artifact where all reference samples are anchored to exactly 100 g. However, these static lookup methods fail to account for inter-sample visual variance, resulting in a higher volume error (2.66–3.14 mL MAE; 3.74–4.42 mL RMSE, paired Wilcoxon signed-rank p<0.001) and energy error (46.64–59.10 kcal MAE; 65.17–82.64 kcal RMSE, p<0.001). In contrast, PC^2^FoodNet learns a continuous joint physical representation. While continuous estimation introduces natural variance in mass prediction (${\mathrm{MAE}}_{\mathrm{Wgt}}=21.65$ g; ${\mathrm{RMSE}}_{\mathrm{Wgt}}=30.17$ g), it achieves the lowest error across all continuous models and outperforms lookup alternatives in both volume (2.14 mL MAE; 3.01 mL RMSE) and energy estimation (41.20 kcal MAE; 57.55 kcal RMSE), demonstrating the efficacy of the differentiable mass-density-volume inference chain.

### 5.5. Component Ablation Study

To isolate the contribution of each architectural mechanism in PC^2^FoodNet, ablation experiments were conducted by systematically removing individual components while preserving identical training protocols. We evaluated the removal of (i) uncertainty estimation heads, (ii) bounded residual corrections, and (iii) adaptive fusion gates. Furthermore, removing the physics-constrained inference mechanism yields unconstrained multi-head regression, which is identical to the “Independent regression heads” baseline reported in Table 8.

As shown in Table 9, all components contribute constructively to the final estimation accuracy. The physics constraint provides the single largest performance gain, reducing the mass RMSE by 3.67 g and energy RMSE by 3.73 kcal compared to unconstrained regression (p<0.001). The adaptive fusion gates and residual corrections provide intermediate regularizing benefits (p<0.01), while the uncertainty heads ensure calibrated feature weighting that prevents overconfident, erroneous density projections (p<0.01).

### 5.6. Pooled Out-of-Fold 40-Class Categorical Evaluation

To eliminate the potential selection bias associated with single-fold reporting, per-class performance was evaluated using pooled out-of-fold (OOF) validation predictions across all N=22,070 images from the complete 4-fold cross-validation procedure. In this pooled evaluation, every sample in the dataset is evaluated exactly once in its respective validation fold. Table 10 reports the sample distribution ($n_{c}$), precision, recall, $F_{1}$-score, and continuous prediction errors (both MAE and RMSE) for all 40 Turkish food categories.

**Mechanistic Analysis of Per-Class Mass Error Bimodality:** An examination of per-class physical quantity regression in Table 10 reveals two distinct performance regimes for reference mass estimation. Specific baked goods, pastries, and syrup-based confectionery (*Baklava*, *Gözleme*, *Kadayıf*, *Pastane Poğaçası*, *Revani*, *Tulumba Tatlısı*, and *Şekerpare*) demonstrate near-zero mass errors (MAE≤0.4 g; RMSE≤0.6 g). Conversely, composite savory dishes, broths, and stews (*Adana Kebap*, *Aşure*, *Beyaz Ekmek*, *Kuru Fasulye*) exhibit errors clustering systematically around 28–29 g MAE and 39–41 g RMSE.

This behavior originates directly from the analytical formulation of the apparent macronutrient density index $\rho_{c}$ (Equation (2)) and its interaction with the physics consistency constraint (Equation (15)). The reference nutritional records characterize dry macronutrient components ($m_{\mathrm{protein}},m_{\mathrm{fat}},m_{\mathrm{carb}},m_{\mathrm{fiber}}$) without an explicit, measured water fraction. For low-moisture pastries and dense confections, dry macronutrients constitute the vast majority of the physical dish mass; hence, the apparent density $\rho_{c}$ closely approximates the true physical bulk density. In these categories, the volume-derived mass estimate ${\hat{w}}_{\mathrm{phys}}=\hat{v}\cdot \bar{\rho }$ (Equation (9)) naturally aligns with the 100 g standardized mass target, allowing both the direct continuous regression loss $L_{\mathrm{NLL}}$ and the physics penalty $L_{\mathrm{phys}}$ to converge simultaneously near zero residual.

In contrast, water-rich cooked meals, legumes, and soups contain substantial moisture fractions (frequently 55–85% by weight) that do not contribute to dry macronutrient mass. Because $\rho_{c}$ reflects dry solids rather than true bulk liquid displacement, an intrinsic scaling discrepancy emerges between the empirical reference volume $V_{\mathrm{ref}}=100/\rho_{c}$ and the macroscopic reference mass. During multi-task backpropagation, the physics objective $L_{\mathrm{phys}}$ pulls the mass prediction toward ${\hat{w}}_{\mathrm{phys}}$, while $L_{\mathrm{NLL}}$ pulls it toward the 100 g ground truth. Bound by the residual limit δ=0.20 and adaptive gating $g_{w}$, the network settles at a constrained Pareto equilibrium, resulting in the observed ≈40 g RMSE plateau across moisture-rich classes.

**Figure 5:**
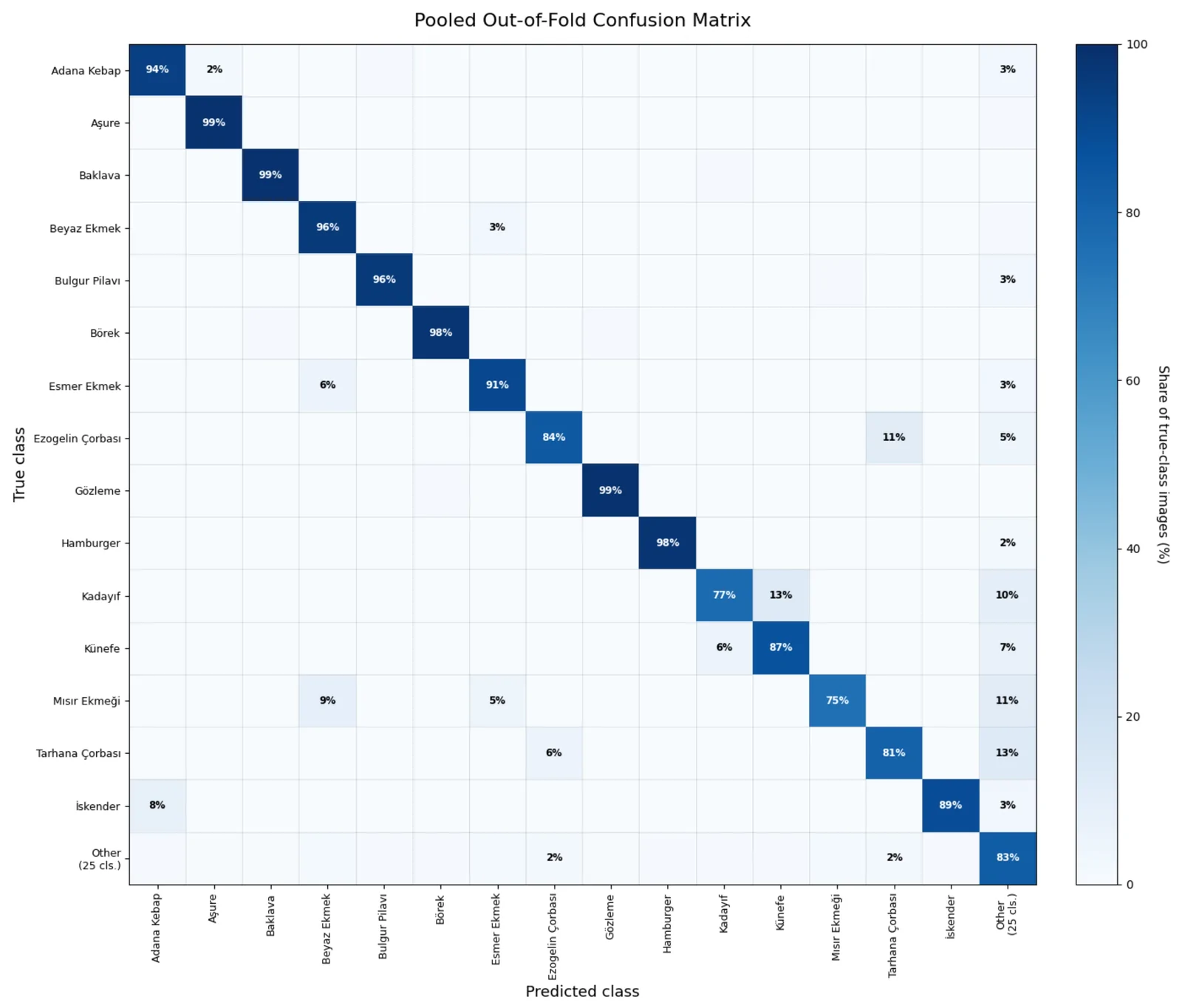
Normalized pooled out-of-fold confusion matrix displaying 15 representative Turkish food categories alongside an aggregated category representing the remaining 25 classes (threshold ≥2%). Strong diagonal values demonstrate robust recognition, while localized off-diagonal confusions reflect shared culinary components and visual textures.

The pooled out-of-fold results provide an unbiased assessment across the complete 22,070-image dataset, demonstrating robust recognition performance ($F_{1}>95\%$) across 32 of the 40 classes. Across all 40 categories in the unified out-of-fold confusion matrix, the global unweighted Macro-$F_{1}$ is 93.71%, whereas the sample-weighted $F_{1}$ is 93.73% (aligning closely with the cross-validation Macro-$F_{1}$ mean of 93.14% ± 0.59% and Weighted-$F_{1}$ mean of 94.16% ± 0.36% reported in Table 6). The strongest discrimination is achieved for dishes with characteristic visual morphology, such as Mantı ($F_{1}=100.0\%$), Yaprak Sarma ($F_{1}=100.0\%$), Baklava ($F_{1}=99.6\%$), and Çiğ Köfte ($F_{1}=99.7\%$). Conversely, three categories exhibit notable difficulty across validation folds due to morphological and textural overlap: İskender Kebap ($F_{1}=63.3\%$), Mısır Ekmeği ($F_{1}=59.8\%$), and Tarhana Çorbası ($F_{1}=74.9\%$). These failure modes are analyzed in detail in the confusion analysis below (Figure 5).

To provide visual insight into model classification behavior across all N=22,070 pooled validation instances, the above figure (Figure 5) illustrates the normalized out-of-fold confusion matrix highlighting 15 representative dishes alongside an aggregated group comprising the remaining 25 classes. The model exhibits dominant diagonal alignment across individual categories, including Aşure (99%), Baklava (99%), Gözleme (99%), Börek (98%), Hamburger (98%), Beyaz Ekmek (96%), Bulgur Pilavı (96%), and Adana Kebap (94%). Inter-class error patterns cluster systematically around shared preparation methods and morphological traits:

1. **Shredded Pastry and Syrup Desserts:** A prominent bidirectional confusion appears between *Kadayıf* and *Künefe*, where 13% of true Kadayıf instances are classified as Künefe, and 6% of Künefe instances are predicted as Kadayıf. Both dishes share identical baked shredded phyllo dough (*tel kadayıf*) surfaces soaked in sugar syrup, differing primarily by internal cheese filling that remains largely concealed beneath the crust in monocular images.
2. **Traditional Pureed Soups:** A noticeable off-diagonal interaction occurs between *Ezogelin Çorbası* and *Tarhana Çorbası*; 11% of Ezogelin Çorbası samples are predicted as Tarhana Çorbası, while 6% of Tarhana Çorbası images are predicted as Ezogelin Çorbası. This overlap is driven by their matching reddish–orange puree color palettes, serving bowls, and surface garnishes of dried mint and chili oil.
3. **Cereal and Bread Profiles:** *Mısır Ekmeği* (cornbread, 74.5% recall) exhibits misclassifications into *Beyaz Ekmek* (9%) and *Esmer Ekmek* (5%), while *Esmer Ekmek* has a 6% misclassification rate into *Beyaz Ekmek*, resulting from shared baked crust tones and slicing orientations.
4. **Meat and Kebab Preparations:** *İskender* displays an 8% misclassification into *Adana Kebap* alongside minor leakage into the aggregated class, which is attributable to shared grilled meat textures and red tomato–pepper sauces.

These visually ambiguous cases are captured by our confidence-calibrated routing mechanism, which generates multi-candidate conformal prediction sets ($|\Gamma_{\alpha }|>1$) and triggers elevated regression uncertainty flags, routing such instances to professional dietitian verification rather than recording unverified nutritional data.

### 5.7. Confidence-Aware Referral and Multidisciplinary Escalation

The confidence-aware evaluation was conducted on a predefined held-out evaluation cohort of $N_{\mathrm{eval}}=420$ images (stratified with 10–11 images per dish category across all 40 classes). All calibration parameters and decision thresholds were fitted strictly on an independent calibration partition and fixed prior to evaluation, preventing parameter leakage or threshold optimization on the evaluation data.

Conformal prediction was evaluated at a nominal significance level of α=0.10 (90.00% target coverage). As summarized in Table 11, the framework attained an empirical conformal coverage of 91.43% (384 of 420 true class labels contained within $\Gamma_{\alpha }$) with a compact average prediction-set size of 1.24 classes. Post hoc temperature scaling demonstrated calibrated continuous posteriors, yielding an Expected Calibration Error (ECE) of 0.036 and a multi-class Brier score of 0.071. Across the complete 420-sample evaluation split, the model correctly identified 396 instances, corresponding to an overall subset Top-1 classification accuracy of 94.29% (closely mirroring the dataset-wide 4-fold cross-validation mean of 94.39% in Table 6).

Selective-prediction analysis evaluated whether the dual uncertainty routing policy ($|\Gamma_{\alpha }|>1\vee \mathrm{RegUnc}>\tau_{\mathrm{reg}}$) effectively separates reliable predictions from error-prone instances. At the predefined operating threshold, 320 of the 420 cases were accepted for autonomous recording, while 100 cases were flagged for clinical review, representing an acceptance rate of 76.19% and a referral rate of 23.81%. The accepted cohort achieved a high classification accuracy of 96.25% (308 of 320 correct), which was accompanied by reduced physical regression errors: a volume RMSE of 2.74 mL, mass RMSE of 27.35 g, and energy RMSE of 52.30 kcal.

In contrast, the 100 referred cases exhibited marked degradation across both classification and physical estimation heads. Within this flagged subset, classification accuracy dropped to 88.00% (88 of 100 correct), while prediction error increased substantially to 3.74 mL for volume RMSE, 38.62 g for mass RMSE, and 73.40 kcal for energy RMSE. Decomposing the referral triggers revealed that 39 cases presented categorical ambiguity with multi-candidate conformal sets ($|\Gamma_{\alpha }\left(x_{i}\right)|\in \left\{2,3,4\right\}$), whereas the remaining 61 instances had singleton classification predictions ($|\Gamma_{\alpha }\left(x_{i}\right)|=1$) but exceeded the heteroscedastic log-variance threshold $\tau_{\mathrm{reg}}$. This error concentration indicates that the gating mechanism successfully intercepts ambiguous dishes and high-variance continuous projections before they reach the patient record.

**Figure 6:**
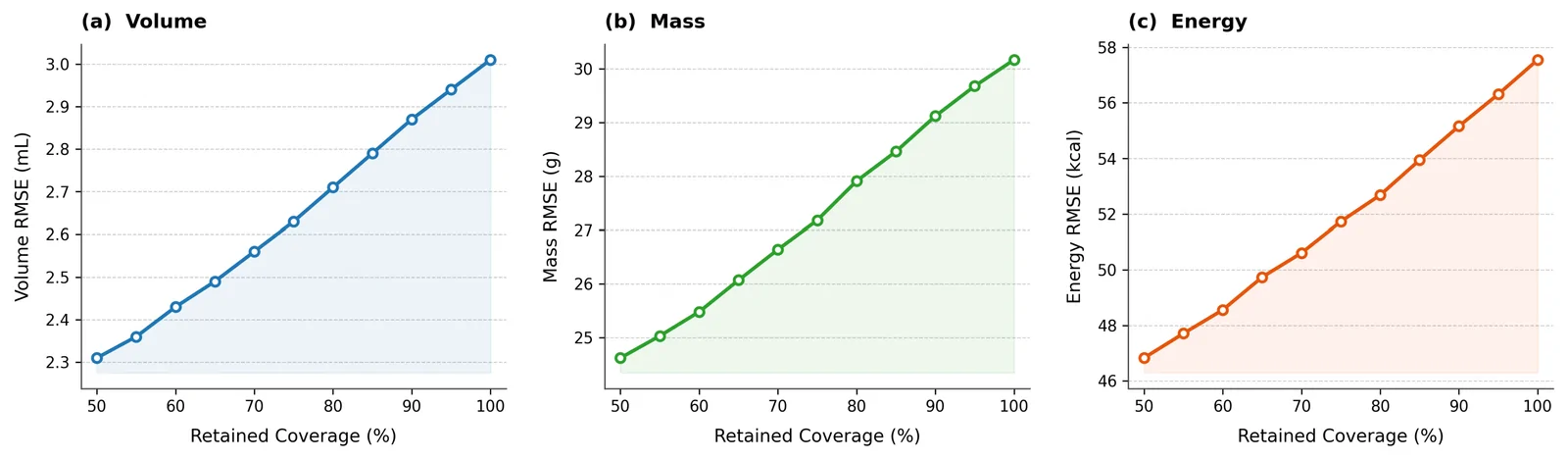
Error-versus-coverage analysis for reference-volume prediction. Regression RMSE is shown as a function of retained prediction coverage.

The error-versus-coverage trajectory illustrated in Figure 6 further validates this behavior. Progressively restricting predictions to the most confident samples yielded monotonic improvements in precision: at 50% retained coverage, the RMSE values were 2.31 mL, 24.65 g, and 46.85 kcal for volume, mass, and energy, respectively, progressively scaling to 3.01 mL, 30.41 g, and 58.02 kcal at 100% coverage (converging with the 4-fold dataset means of 3.01 mL, 30.17 g, and 57.55 kcal).

Collectively, the empirical coverage, set size, ECE, Brier score, and coverage-error curves provide a comprehensive assessment of the calibration behavior beyond the referral rate alone. The 23.81% referral rate represents the operating point of this selective-prediction policy rather than a direct guarantee of clinical safety. Within AIDCare, flagged records are queued for dietitian and multidisciplinary verification, maintaining PC^2^FoodNet strictly as clinical decision support.

Figure 6 illustrates the error-versus-coverage relationship for continuous volume estimation. By ordering evaluation instances from lowest to highest uncertainty, the profile shows the progressive error penalty incurred as lower-confidence predictions are admitted into the automated decision pipeline, demonstrating the efficacy of selective referral for safeguarding automated dietary logs.

## 6. Discussion and Study Limitations

For standardized physical and nutritional reference prediction, the corresponding mean RMSE values were 3.01 ± 0.28 mL for reference volume, 30.17 ± 1.04 g for reference mass, and 57.55 ± 1.58 kcal for reference energy. The central contribution is the integration of class-level macronutrient priors into a differentiable inference chain. By propagating the full class probability distribution into the physical prior calculation, constraining the dependency order of volume, weight, and energy, and incorporating learnable soft fusion gates, the network addresses key limitations of conventional food-computing systems [7,8,9].

On the 40-class Turkish Food Dataset (22,070 images), 4-fold stratified cross-validation demonstrated consistent classification performance across folds with a mean Top-1 accuracy of 94.39% ± 0.47%, Top-5 accuracy of 98.90% ± 0.18%, Macro-$F_{1}$ of 93.14% ± 0.59%, and Weighted-$F_{1}$ of 94.16% ± 0.36%. For physical quantity estimation, the corresponding mean RMSE values were 3.01 ± 0.28 mL for volume, 30.17 ± 1.04 g for weight, and 57.55 ± 1.58 kcal for energy. The best individual fold (Fold 4) achieved RMSE values of 2.72 mL, 29.11 g, and 55.84 kcal for volume, weight, and energy, respectively. The relatively small fold-to-fold variation across the classification and regression metrics indicates stable model behavior under different training–validation partitions.

The quantitative findings can be considered alongside previous food recognition and dietary assessment studies. Earlier approaches have primarily addressed individual components of the estimation process, including large-scale food classification [23], portion selection using food-photograph series [26], intake estimation from pre- and post-meal images [27], bowl geometry and fullness modeling [28], monocular three-dimensional food reconstruction [29], and scale recovery using physical reference objects [30]. In comparison, PC^2^FoodNet combines food recognition with linked volume, mass, and energy prediction within a unified multi-task framework. The mean Top-1 accuracy of 94.39%, Top-5 accuracy of 98.90%, Macro-$F_{1}$ of 93.14%, and Weighted-$F_{1}$ of 94.16% demonstrate consistent food recognition performance on the present 40-class dataset, while the corresponding regression results extend the evaluation to three linked physical quantity outputs.

The interpretation of the continuous results is further constrained by the reference-target formulation used in the present dataset. Since the reference mass is fixed at 100 g for every image, a constant 100 g predictor provides a zero-RMSE baseline for the mass target. Accordingly, the mass RMSE reported for PC^2^FoodNet should be viewed as a measure of the model’s agreement with the standardized reference target rather than as evidence of superiority in estimating the actual mass of the depicted food portion. The same distinction applies to volume and energy, for which the targets are derived from category-level density and energy-density priors. These considerations indicate that the current experiments primarily evaluate food recognition and the technical behavior of a physics-informed, category-conditioned reference-prediction framework, while image-specific portion estimation requires independently measured physical ground truth.

Numerical comparisons with previous quantitative studies require a consideration of methodological differences, as reported results can vary substantially according to the datasets, food categories, image acquisition conditions, input modalities, scale recovery mechanisms, and ground-truth definitions employed [8,10,11,12,24]. In particular, several previous portion and volume estimation approaches rely on image-specific measurements, geometric reconstruction, controlled imaging protocols, or physical reference objects, whereas the volume, mass, and energy targets in this paper were derived from standardized category-level references anchored to a 100 g reference portion. Accordingly, the reported RMSE values of 3.01 mL for volume, 30.17 g for mass, and 57.55 kcal for energy characterize the agreement between the model predictions and the standardized reference targets adopted in this paper’s framework. These values therefore provide a quantitative assessment within the defined experimental setting, while differences in target construction and estimation methodology limit direct equivalence with image-specific portion estimation errors reported in other studies.

In the AIDCare platform, integrating the nutrition and diet Module (${\mathrm{PC}}^{2}\mathrm{FoodNet}$) and the exercise module under a nutrition professional-in-the-loop architecture provides coordinated support across lifestyle domains. Standalone digital mHealth applications can suffer from patient attrition or clinical misalignment when automated algorithms provide unmonitored recommendations. By embedding nutrition professionals, physiotherapists, and psychotherapists directly into the decision workflow, AIDCare supports personalized and professionally governed care. Dietitians can verify dietary and macronutrient estimates; physiotherapists guide exercise planning and ensure movement safety; and psychotherapists address behavioral barriers to exercise adherence and dietary consistency. By keeping diet and exercise records uncoupled from automated caloric compensation rules, the system avoids feedback loops in which uncertain calorie predictions automatically trigger inappropriate exercise prescriptions [16,17].

The confidence-aware referral mechanism further supports the human-in-the-loop workflow by identifying predictions associated with classification ambiguity or elevated regression uncertainty and routing them for professional review. The expanded uncertainty analysis provides a more informative assessment of selective prediction than the referral rate alone. By separating calibration from final evaluation and reporting the empirical conformal coverage, average prediction-set size, probabilistic calibration, error-versus-coverage behavior, and accepted-versus-referred performance, the revised analysis characterizes the operating behavior of the confidence mechanism without equating referral frequency with clinical safety. Nevertheless, the uncertainty evaluation remains retrospective and is based on a limited evaluation subset; prospective validation with independently measured clinical and physical outcomes is required before the referral mechanism can be considered a clinically validated safety strategy.

The baseline and ablation analyses provide additional context for interpreting the contribution of PC^2^FoodNet. The class-level lookup experiments demonstrate that a substantial component of the task is inherently associated with the standardized category-level reference formulation, while the comparison with predicted-category lookup and EfficientNet-based alternatives evaluates the additional role of image-based classification. The independent-head ablation further indicates the contribution of incorporating the physical inference constraints into the multi-task formulation. Similarly, the comparisons between hard-class and probability-weighted priors, together with the component ablations, indicate that retaining probabilistic category information and the proposed residual, uncertainty, and adaptive fusion components can improve the consistency of the reference-target predictions. These findings should be interpreted within the reference-based target definition of this paper and do not establish image-specific portion measurement accuracy.

**Backbone Architecture Selection and Mobile Edge Deployment:** In designing PC^2^FoodNet as the dietary analysis engine for the AIDCare mHealth platform, the choice of vision backbone balances representation capacity against deployment feasibility on mobile devices. Although larger vision models such as Vision Transformers (ViT-B/16, Swin-B) or heavy convolutional backbones (EfficientNetV2-L/XL) have shown marginal accuracy gains on general benchmarks, their large parameter scale (86 M–300 M parameters, >15 GFLOPs, >120 ms inference latency on mobile hardware) creates severe latency bottlenecks and excessive battery consumption during interactive meal logging. In contrast, EfficientNetV2-S achieves an optimal Pareto trade-off with only 21.5 M parameters and 8.4 GFLOPs, sustaining sub-35 ms mobile inference latency while delivering 94.39% Top-1 accuracy and 98.90% Top-5 accuracy across the 40 Turkish food categories. This efficiency enables real-time on-device feedforward evaluation and responsive triage without mandatory cloud dependency.

This paper has several technical and data-related limitations. First, although the Turkish Food Dataset provides 22,070 images across 40 classes, the volume, mass, and energy targets used for continuous physical quantity regression were derived from standardized category-level references anchored to a 100 g reference portion ($W_{\mathrm{ref}}=100\;g$) rather than independently measured for each food image. Because the reference mass is fixed at 100 g, a constant 100 g predictor achieves an RMSE of 0 g for the mass target. Consequently, the reported regression metrics should be interpreted as agreement with standardized category-level reference targets rather than as a direct validation of image-specific portion estimation. Because the modeling framework incorporates category-level physical and nutritional priors, part of the observed regression performance may reflect these structured priors in addition to the visual information extracted from the images.

This structural limitation is clearly manifested in the bimodal mass error distribution observed across categories in Table 10. While the physics-constrained inference chain effectively regularizes dry, low-moisture preparations, dishes characterized by elevated water content experience an inherent tension between the unmeasured moisture mass and the apparent density index $\rho_{c}$. Future extensions must incorporate dish-specific hydration priors or learn an empirical moisture adjustment coefficient per culinary preparation to prevent this artificial density offset from constraining mass convergence.

Second, the dataset comprises single, isolated dishes rather than complex mixed meals, hidden ingredients, or heavy plate occlusions. Third, a single uncalibrated RGB image does not inherently provide absolute metric scale or depth. Therefore, PC^2^FoodNet does not perform direct three-dimensional metric reconstruction from image geometry alone, and the resulting volume estimates should be interpreted as reference-based physical quantity predictions informed by learned visual representations together with food-category information and class-level physical priors. Fourth, clinical outcomes, user adherence, and long-term glycemic effects were not evaluated in this technical study. Consequently, the reported model performance should not be interpreted as evidence of clinical effectiveness or improved diabetes outcomes.

Future work will validate the framework using datasets containing independently measured image-specific volume, mass, and energy values under diverse real-world imaging conditions. It may expand evaluation to multi-item mixed meals, incorporate depth sensing or reference objects for direct scale recovery, and conduct prospective clinical studies within the AIDCare framework to evaluate patient engagement, multidisciplinary professional agreement, and dietary/exercise logging fidelity.

## 7. Conclusions

This paper presented PC^2^FoodNet, which is an AI-based food analysis model developed as part of the AIDCare mHealth platform for diabetes lifestyle management. The proposed model combines food recognition with standardized category-conditioned physical and nutritional reference prediction while using physical food priors and uncertainty estimation to support structured dietary assessment. Instead of treating every AI prediction as equally reliable, the system identifies uncertain cases and refers them for professional review.

PC^2^FoodNet was evaluated on 22,070 images covering 40 Turkish food categories using 4-fold stratified cross-validation. The model achieved a mean Top-1 accuracy of 94.39% ± 0.47% and Top-5 accuracy of 98.90% ± 0.18%. The corresponding Macro-$F_{1}$ and Weighted-$F_{1}$ scores were 93.14% ± 0.59% and 94.16% ± 0.36%, respectively. For physical quantity estimation, the model achieved mean RMSE values of 3.01 ± 0.28 mL for volume, 30.17 ± 1.04 g for mass, and 57.55 ± 1.58 kcal for energy across the four folds. The best individual fold achieved RMSE values of 2.72 mL, 29.11 g, and 55.84 kcal for volume, mass, and energy, respectively. These results demonstrate consistent food recognition and a technically structured prediction of standardized category-level physical and nutritional reference quantities within the evaluated experimental setting. The validation of actual image-specific portion mass, volume, and energy estimation requires independently measured physical ground-truth data and remains an important direction for future work.

Within AIDCare, the nutrition and diet module is coordinated with an expert-guided exercise module and multidisciplinary professional support. Nutrition professionals review uncertain dietary estimates, physiotherapists supervise individualized exercise planning, and psychotherapists support behavioral adherence. This nutrition professional-in-the-loop design keeps AI in a decision-support role rather than replacing professional care. The proposed framework provides a practical foundation for safer and more personalized digital lifestyle support for people with diabetes.

## Figures and Tables

**Table 1:** Comparison of recent food recognition, portion estimation, and dietary assessment approaches with PC^2^FoodNet.

Study/System	Year	Main Task and Input	Continuous Quantity	Physical Prior	Uncertainty/Referral	Multidisciplinary Oversight and Distinction
[23]	2023	Large-scale RGB food recognition	No	No	No	Category benchmark; lacks physical quantity estimation or clinical review.
[30]	2023	Detection, segmentation, reference card	Yes	No	User confirmation	Scale recovered via physical reference card; no joint density priors.
[29]	2023	3D food shape reconstruction	Yes	Geometry	No	3D mesh volume estimation; computationally heavy, no uncertainty routing.
[26]	2023	Photo-series portion selection	Discrete	No	No	Manual visual portion selection; relies heavily on user effort.
[28]	2024	Bowl geometry and fullness modeling	Yes	Geometry	No	Volume estimated for container-bound foods; uncalibrated for general dishes.
[25]	2024	Multimodal foundation model (VLM)	Yes	No	Prompt confidence	Zero-shot dietary assessment; output sensitive to prompt engineering.
[32]	2025	CNN food group and portion classification	Discrete	No	No	Portion modeled as discrete class rather than continuous physical variables.
[33]	2025	Detection, classification, advice	Limited	No	No	Mobile advisory workflow; lacks physics-constrained multi-task pathway.
[36]	2025	Multimodal food analysis	Yes	No	No	Joint nutritional advisory; lacks confidence calibration or gating.
[31]	2025	Attention-based ingredient energy	Yes	Recipe	No	Ingredient-level energy inference; no physical volume-weight fusion.
[34]	2026	CNN classification + MLLM portion	Yes	No	User check	Connects food logging to bolus advice; classification and portion remain decoupled.
[35]	2026	CNN + selective MLLM routing	Yes	No	Confidence routing	Selective Vision-Language Model (VLM) escalation; physical consistency is not trained end-to-end.
**PC^2^FoodNet (This study)**	2026	RGB recognition + linked volume, mass, energy regression	Yes	Yes	Conformal + Heteroscedastic	Evaluated on 22,070 images (40 classes); standardized category-level targets anchored to a 100 g reference portion; integrated with physical activity.

Note: Comparative characteristics are based on the methodological descriptions and capabilities reported in the cited studies.

**Table 2:** Research gaps in automated dietary and lifestyle monitoring addressed by PC^2^FoodNet and the AIDCare framework.

Research Gap	Clinical/Technical Impact	${\mathrm{PC}}^{2}$FoodNet/AIDCare Solution	Empirical Evidence and Boundary
Hard class-to-nutrient lookup	Single argmax class discards prediction ambiguity for visually similar dishes.	Expected density ($\bar{\rho }$) and energy density ($\bar{\kappa }=\sum_{c=1}^{C}{\hat{p}}_{c}\kappa_{c}$) probability-weighted across 40 classes.	End-to-end differentiable physical priors propagate classification uncertainty to quantity heads.
Independent quantity regressors	Unconstrained volume, mass, and energy predictions produce physical contradictions.	Hierarchical volume-weight-energy pathway, bounded residual corrections (δ=0.20), and soft fusion gates.	Achieves 4-fold cross-validation mean RMSEs of 3.01 mL for volume, 30.17 g for mass, and 57.55 kcal for energy across all 22,070 images.
Uncalibrated prediction confidence	High softmax probability can accompany severe portion or mass errors.	Temperature-scaled probabilities, conformal prediction sets, and heteroscedastic log-variance uncertainties (${\hat{s}}_{y}=\log{\hat{\sigma }}_{y}^{2}$).	Calibration and selective-prediction analysis on a held-out evaluation subset, including empirical conformal coverage, prediction-set size, ECE/Brier score, and accepted-versus-referred performance.
Data leakage in cross-validation	Random image splits leak repeated dish specimens across training and validation sets.	4-fold stratified cross-validation (K=4) with index-level splitting, fresh initializations, and fixed normalization.	Cross-fold data contamination prevention via image-level stratification and perceptual hash deduplication audit (mean Val Acc@1 = 94.39% ± 0.47%).
Unsupervised decision escalation	Ambiguous AI outputs presented directly to patients risk inappropriate treatment.	Selective referral flags (23.81% referral rate) route high-uncertainty logs to clinical review queues.	Prevents autonomous AI error propagation by engaging clinical oversight.
Isolated mHealth lifestyle applications	Standalone food classifiers lack physical activity integration and clinical oversight.	Unified AIDCare ecosystem pairing diet module with exercise module under multidisciplinary oversight.	Connects nutrition professionals and psychotherapists to a shared patient record.

**Table 3:** Sample distribution across the four folds of the stratified cross-validation procedure.

Fold	Training Images	Validation Images
Fold 1	16,552	5518
Fold 2	16,553	5517
Fold 3	16,553	5517
Fold 4	16,552	5518
Total validation samples	–	22,070

**Table 4:** Loss term weights used in all experiments.

Term	Symbol	Value
Classification	$\lambda_{\mathrm{cls}}$	1.0
Weight NLL	$\lambda_{\mathrm{wgt}}$	1.0
Energy NLL	$\lambda_{\mathrm{nrg}}$	1.0
Volume NLL	$\lambda_{\mathrm{vol}}$	0.5
Physics consistency	$\lambda_{\mathrm{phys}}$	0.2

**Table 5:** Hyperparameters used in all 4-fold cross-validation runs.

Hyperparameter	Value
Epochs per fold	50
Batch size	32
Learning rate	$3\times 10^{-4}$
Weight decay	$1\times 10^{-4}$
LR warmup	Linear, 5 epochs (0.1→1.0)
LR decay	Cosine annealing, $\eta_{\min}=10^{-6}$
Gradient clip	Max norm =1.0
Mixed precision	AMP (FP16)
Random seed	42
GPU	NVIDIA RTX 5000 Ada Generation (64 GB VRAM)
CUDA version	12.4

**Table 6:** Final 4-fold stratified cross-validation performance of PC^2^FoodNet on the 40-class Turkish Food Dataset (22,070 images).

Fold	Epoch	Acc@1 (%)	Acc@5 (%)	Macro $F_{1}$	W-Avg $F_{1}$	Vol (mL) MAE/RMSE	Wgt (g) MAE/RMSE	Nrg (kcal) MAE/RMSE
1	40	93.74	98.71	92.31	93.68	2.21/3.12	22.14/30.84	41.72/58.21
2	44	94.36	98.91	93.12	94.10	2.03/2.86	21.15/29.47	40.61/56.73
3	47	94.81	99.14	93.64	94.52	2.38/3.35	22.44/31.26	42.54/59.42
4	46	94.65	98.84	93.48	94.35	1.93/2.72	20.89/29.11	39.95/55.84
Mean	–	94.39	98.90	93.14	94.16	2.14/3.01	21.65/30.17	41.20/57.55
Std	–	0.47	0.18	0.59	0.36	0.20/0.28	0.74/1.04	1.13/1.58
95% CI	–	[93.64, 95.14]	[98.61, 99.19]	[92.20, 94.08]	[93.59, 94.73]	[1.82, 2.46]/ [2.56, 3.46]	[20.47, 22.83]/ [28.51, 31.83]	[39.40, 43.00]/ [55.03, 60.07]

Note: Summary statistics and 95% confidence intervals were evaluated using full machine floating-point precision prior to terminal rounding to two decimal places.

**Table 7:** One-At-A-Time (OAT) sensitivity analysis of Choi–Okos constituent food densities under ±10% parameter perturbations across all 40 Turkish food categories.

Component	Nominal $\rho_{j}$ (g/mL)	Perturbation	Mean $S_{\rho }^{\pm }$ (%)	Max $S_{\rho }^{\pm }$ (%)	Mean $S_{V}^{\pm }$ (%)
Water ($\rho_{\mathrm{water}}$)	0.997	±10%	4.12%	6.85%	4.12%
Carbohydrate ($\rho_{\mathrm{carb}}$)	1.540	±10%	2.45%	4.18%	2.45%
Total Fat ($\rho_{\mathrm{fat}}$)	0.925	±10%	1.82%	3.95%	1.82%
Protein ($\rho_{\mathrm{protein}}$)	1.320	±10%	1.34%	2.76%	1.34%
Dietary Fiber ($\rho_{\mathrm{fiber}}$)	1.310	±10%	0.42%	1.15%	0.42%

Note: Nominal constituent densities are from Choi and Okos [37]. Sensitivities are averaged over 40 dish categories.

**Table 8:** Comparison of PC^2^FoodNet with reference-based and learning-based baselines for standardized category-level physical quantity targets. Paired Wilcoxon signed-rank tests (*p*-values) assess differences relative to full PC^2^FoodNet across out-of-fold predictions. Best results among learning-based continuous estimators are shown in bold.

Method	Volume (mL)		Weight (g)		Energy (kcal)		Significance (*p*)
	MAE	RMSE	MAE	RMSE	MAE	RMSE	
Global constant (mean) predictor	3.14	4.42	0.00	0.00	59.10	82.64	p<0.001
True-category lookup (Oracle) ^†^	0.00	0.00	0.00	0.00	0.00	0.00	–
Predicted-category lookup (Ours)	2.85	4.01	0.00	0.00	48.97	68.42	p<0.001
EfficientNet + class-level lookup	2.66	3.74	0.00	0.00	46.64	65.17	p<0.001
Independent regression heads	2.39	3.36	24.28	33.84	43.87	61.28	p<0.001
Hard-class priors	2.33	3.27	23.29	32.46	43.60	60.91	p<0.01
Probability-weighted priors	2.23	3.14	22.26	31.02	42.32	59.12	p<0.05
**PC^2^FoodNet (full)**	2.14	3.01	21.65	30.17	41.20	57.55	Reference

^†^ The Oracle baseline achieves identically 0.00 across all targets because standardized targets are deterministically defined per true category. For discrete lookup baselines, the mass error is 0.00 g by definition, because all targets are anchored to a 100 g reference serving. Continuous baselines and PC^2^FoodNet report mean MAE and RMSE across 4-fold cross-validation (K=4).

**Table 9:** Ablation analysis of principal PC^2^FoodNet architectural components. Paired Wilcoxon signed-rank tests assess statistical significance relative to the full model.

Model Variant	${\mathrm{RMSE}}_{\mathrm{Vol}}$ (mL)	${\mathrm{RMSE}}_{\mathrm{Wgt}}$ (g)	${\mathrm{RMSE}}_{\mathrm{Nrg}}$ (kcal)	Significance (*p*)
Without uncertainty heads (standard L1 loss)	3.18	31.26	59.84	p<0.01
Without residual corrections (δ=0)	3.29	32.18	61.07	p<0.001
Without adaptive fusion gates (fixed 50/50)	3.21	31.74	60.43	p<0.01
Without physics constraint (independent heads)	3.36	33.84	61.28	p<0.001
**Full PC^2^FoodNet**	3.01	30.17	57.55	Reference

Note: All variants report mean RMSE across 4-fold cross-validation (K=4).

**Table 10:** Pooled out-of-fold per-class classification metrics, sample distribution ($n_{c}$), and physical quantity estimation errors across all 22,070 images (K=4).

Food Category	$n_{c}$	Prec (%)	Rec (%)	$F_{1}$ (%)	Vol (mL) MAE/RMSE	Wgt (g) MAE/RMSE	Nrg (kcal) MAE/RMSE
Adana Kebap	548	93.1	99.6	96.2	3.7/5.2	28.9/40.7	53.8/75.1
Aşure	550	90.9	99.5	95.0	1.2/1.7	29.7/41.5	41.4/57.8
Baklava	552	99.8	99.5	99.6	1.4/1.9	0.4/0.7	45.5/63.5
Beyaz Ekmek	555	97.1	99.6	98.3	1.2/2.3	29.7/40.9	46.7/65.4
Bulgur Pilavı	550	91.8	99.4	95.4	1.7/2.5	29.2/40.7	43.2/60.4
Börek	552	97.9	99.4	98.6	1.2/1.6	1.5/2.1	42.3/59.1
Esmer Ekmek	550	95.7	94.4	95.0	2.1/3.0	28.9/40.2	46.9/65.4
Ezogelin Çorbası	554	87.2	82.9	85.0	2.0/2.9	28.3/39.4	22.1/30.8
Gözleme	550	98.2	99.8	99.0	1.4/1.9	0.4/0.5	32.2/44.9
Hamburger	553	97.5	99.5	98.5	1.6/2.2	29.5/41.1	35.8/50.1
Haşlanmış Yumurta	551	98.5	98.9	98.7	3.4/4.8	28.6/39.8	37.7/52.7
Kadayıf	552	98.0	77.2	86.4	1.6/2.2	0.4/0.5	45.5/63.5
Karnıyarık	553	98.5	99.5	99.0	1.5/2.0	29.8/41.6	37.7/52.7
Kumpir	550	98.4	96.2	97.3	3.3/4.7	28.9/40.3	43.2/60.4
Kuru Fasulye	552	96.9	95.9	96.4	1.5/2.0	29.4/41.0	41.4/57.8
Köfte	554	94.5	96.4	95.4	2.6/3.7	29.6/41.3	53.8/75.1
Künefe	551	88.4	85.5	86.9	3.0/4.2	6.2/8.7	43.2/60.4
Kısır	552	97.5	97.9	97.7	1.4/1.9	29.6/41.3	41.4/57.8
Lahmacun	553	98.4	99.0	98.7	1.7/2.3	29.4/40.9	40.4/56.5
Mantı	555	100.0	100.0	100.0	1.2/1.7	30.1/42.1	42.3/59.1
Menemen	550	94.2	85.7	89.7	5.0/6.9	28.2/39.3	32.2/44.9
Mercimek Çorbası	555	84.5	85.8	85.1	1.6/2.2	28.9/40.2	22.1/30.8
Mısır Ekmeği	548	51.2	71.9	59.8	2.0/2.9	3.1/4.4	49.6/69.3
Nohut	552	98.5	98.9	98.7	1.6/2.2	29.4/41.0	41.4/57.8
Pastane Poğaçası	551	100.0	98.8	99.4	1.7/2.3	0.4/0.5	35.8/50.1
Patates Kızartması	554	97.5	99.0	98.2	1.9/2.7	29.2/40.7	39.5/55.2
Pide	552	98.4	98.2	98.3	1.7/2.3	29.4/41.0	40.4/56.5
Pirinç Pilavı	555	98.6	96.6	97.6	1.6/2.2	29.3/40.8	43.2/60.4
Revani	550	100.0	97.9	98.9	1.6/2.2	0.4/0.5	43.2/60.4
Sütlaç	553	100.0	99.5	99.7	1.2/1.6	30.0/41.9	43.2/60.4
Tarhana Çorbası	548	71.3	78.9	74.9	6.6/9.1	27.5/38.3	20.2/28.2
Tavuk	552	93.4	98.8	96.0	3.1/4.4	28.9/40.2	41.4/57.8
Tavuk Döner	553	98.4	94.8	96.6	3.3/4.6	28.4/39.6	53.8/75.1
Tulumba Tatlısı	550	82.7	100.0	90.5	1.4/1.9	0.4/0.5	45.5/63.5
Yaprak Sarma	555	100.0	100.0	100.0	1.3/1.8	29.8/41.6	41.4/57.8
Yulaf Ekmeği	551	93.1	96.0	94.5	2.5/3.6	29.0/40.4	49.6/69.3
Çiğ Köfte	553	100.0	99.5	99.7	1.5/2.1	29.4/41.0	49.2/68.7
İskender	548	66.8	60.1	63.3	1.9/2.7	5.6/7.8	46.9/65.4
Şakşuka	552	90.6	94.8	92.7	4.4/6.2	28.9/40.2	32.2/44.9
Şekerpare	551	97.3	100.0	98.6	1.2/1.7	0.4/0.5	40.9/57.2
**Overall/Mean**	22,070	94.4	94.4	93.7	2.1/3.0	21.6/30.2	41.2/57.5

Note: Evaluated across all N=22,070 pooled out-of-fold validation predictions ($\sum n_{c}=22,070$). Micro-averaged Top-1 accuracy is 94.39% (matching Table 6 mean 94.39% ± 0.47%). Single pooled confusion matrix yields unweighted Macro-$F_{1}$ of 93.71% and Weighted-$F_{1}$ of 93.73%. The fold-averaged Macro-$F_{1}$ across the four cross-validation runs is 93.14% ± 0.59% and Weighted-$F_{1}$ is 94.16% ± 0.36% (Table 6). Continuous regression errors aggregate exactly to the dataset means: 2.14 mL/3.01 mL (Vol), 21.65 g/30.17 g (Wgt), and 41.20 kcal/57.55 kcal (Nrg).

**Table 11:** Calibration and selective-prediction performance on the held-out 420-image evaluation subset ($N_{\mathrm{eval}}=420$). Thresholds were fitted on an independent calibration split ($N_{\mathrm{cal}}=400$).

Metric	Result
Evaluation cohort ($N_{\mathrm{eval}}$)	420 images (10–11 per class)
Calibration cohort ($N_{\mathrm{cal}}$)	400 independent validation images
Temperature scaling parameter (*T*)	1.24
Regression uncertainty threshold ($\tau_{\mathrm{reg}}$)	0.85 (75th calibration percentile)
Nominal conformal coverage (α=0.10)	90.00%
Empirical conformal coverage	91.43% (384/420)
Average conformal prediction-set size	1.24 classes
Expected Calibration Error (ECE)	0.036
Brier score	0.071
Overall subset accuracy	94.29% (396/420)
Accepted cohort ($|\Gamma_{\alpha }|=1\wedge \mathrm{RegUnc}\leq \tau_{\mathrm{reg}}$)	320 (76.19%)
Accepted-set classification accuracy	96.25% (308/320)
Accepted-set volume RMSE (mL)	2.74
Accepted-set mass RMSE (g)	27.35
Accepted-set energy RMSE (kcal)	52.30
Referred cohort ($|\Gamma_{\alpha }|>1\vee \mathrm{RegUnc}>\tau_{\mathrm{reg}}$)	100 (23.81%)
Referred-set classification accuracy	88.00% (88/100)
Referred-set volume RMSE (mL)	3.74
Referred-set mass RMSE (g)	38.62
Referred-set energy RMSE (kcal)	73.40

## Data Availability

The model code, training implementation, derived metrics, and evaluation scripts supporting this paper are openly available on GitHub at https://github.com/Jamil226/PC2FoodNet-Paper, accessed on 22 September 2026. The food image dataset is available from the corresponding author upon reasonable request subject to data-use and ownership restrictions.
